# Updating standards for reporting diagnostic accuracy: the development of STARD 2015

**DOI:** 10.1186/s41073-016-0014-7

**Published:** 2016-06-07

**Authors:** Daniël A. Korevaar, Jérémie F. Cohen, Johannes B. Reitsma, David E. Bruns, Constantine A. Gatsonis, Paul P. Glasziou, Les Irwig, David Moher, Henrica C. W. de Vet, Douglas G. Altman, Lotty Hooft, Patrick M. M. Bossuyt

**Affiliations:** 1 Department of Clinical Epidemiology, Biostatistics and Bioinformatics, Academic Medical Centre, University of Amsterdam, Amsterdam, The Netherlands; 2 INSERM UMR 1153 and Department of Pediatrics, Necker Hospital, AP-HP, Paris Descartes University, Paris, France; 3 Julius Center for Health Sciences and Primary Care, University Medical Center Utrecht, University of Utrecht, Utrecht, The Netherlands; 4 Department of Pathology, University of Virginia School of Medicine, Charlottesville, VA USA; 5 Center for Statistical Sciences, Brown University School of Public Health, Providence, RI USA; 6 Centre for Research in Evidence-Based Practice, Faculty of Health Sciences and Medicine, Bond University, Gold Coast, Queensland Australia; 7 Screening and Diagnostic Test Evaluation Program, School of Public Health, University of Sydney, Sydney, New South Wales Australia; 8 Clinical Epidemiology Program, Ottawa Hospital Research Institute, Ottawa, Canada; 9 School of Epidemiology, Public Health and Preventive Medicine, University of Ottawa, Ottawa, Canada; 10 Department of Epidemiology and Biostatistics, EMGO Institute for Health and Care Research, VU University Medical Center, Amsterdam, The Netherlands; 11 Centre for Statistics in Medicine, Nuffield Department of Orthopaedics, Rheumatology and Musculoskeletal Sciences, University of Oxford, Oxford, UK; 12 Dutch Cochrane Centre, Julius Center for Health Sciences and Primary Care, University Medical Center Utrecht, University of Utrecht, Utrecht, The Netherlands

**Keywords:** STARD, Diagnostic accuracy, Sensitivity and specificity, EQUATOR, CONSORT, Reporting quality, Research waste

## Abstract

**Background:**

Although the number of reporting guidelines has grown rapidly, few have gone through an updating process. The STARD statement (Standards for Reporting Diagnostic Accuracy), published in 2003 to help improve the transparency and completeness of reporting of diagnostic accuracy studies, was recently updated in a systematic way. Here, we describe the steps taken and a justification for the changes made.

**Results:**

A 4-member Project Team coordinated the updating process; a 14-member Steering Committee was regularly solicited by the Project Team when making critical decisions. First, a review of the literature was performed to identify topics and items potentially relevant to the STARD updating process. After this, the 85 members of the STARD Group were invited to participate in two online surveys to identify items that needed to be modified, removed from, or added to the STARD checklist. Based on the results of the literature review process, 33 items were presented to the STARD Group in the online survey: 25 original items and 8 new items; 73 STARD Group members (86 %) completed the first survey, and 79 STARD Group members (93 %) completed the second survey.Then, an in-person consensus meeting was organized among the members of the Project Team and Steering Committee to develop a consensual draft version of STARD 2015. This version was piloted in three rounds among a total of 32 expert and non-expert users. Piloting mostly led to rewording of items. After this, the update was finalized. The updated STARD 2015 list now consists of 30 items. Compared to the previous version of STARD, three original items were each converted into two new items, four original items were incorporated into other items, and seven new items were added.

**Conclusions:**

After a systematic updating process, STARD 2015 provides an updated list of 30 essential items for reporting diagnostic accuracy studies.

**Electronic supplementary material:**

The online version of this article (doi:10.1186/s41073-016-0014-7) contains supplementary material, which is available to authorized users.

## Background

The STARD statement (Standards for Reporting Diagnostic Accuracy) was published in 2003. It was intended to help improve the transparency and completeness of reporting of diagnostic accuracy studies. STARD presented a checklist of 25 items that authors should address when reporting diagnostic accuracy studies [1, 2].

Since its publication, STARD has been adopted by more than 200 biomedical journals [3]. Evaluations of adherence to STARD have revealed statistically significant but modest improvements over time in the reporting of diagnostic accuracy studies [4–6]. Unfortunately, reporting remains inadequate for many studies, and journals differ in the extent to which they endorse STARD, recommend it to authors, and use it in the editorial and peer-review process [7–10].

STARD had not been updated in the first 10 years of its existence. In February 2013, the STARD Steering Committee agreed that an update was justified to achieve two main goals (1) to include new items, based on improved understanding of sources of bias and variability, and (2) to facilitate the use of the list, by rearranging and rephrasing existing items, and by improving consistency in wording with other major reporting guidelines such as CONSORT (Consolidated Standards of Reporting Trials) [11].

Although the number of reporting guidelines has grown rapidly, few have gone through an updating process [12]. In this paper, we describe the steps taken to update the original STARD statement, resulting in STARD 2015 [13], and provide a justification for the changes made. The description of our methods may serve as guidance for other groups considering updates of their reporting guidelines.

## Methods

**Fig. 1:**
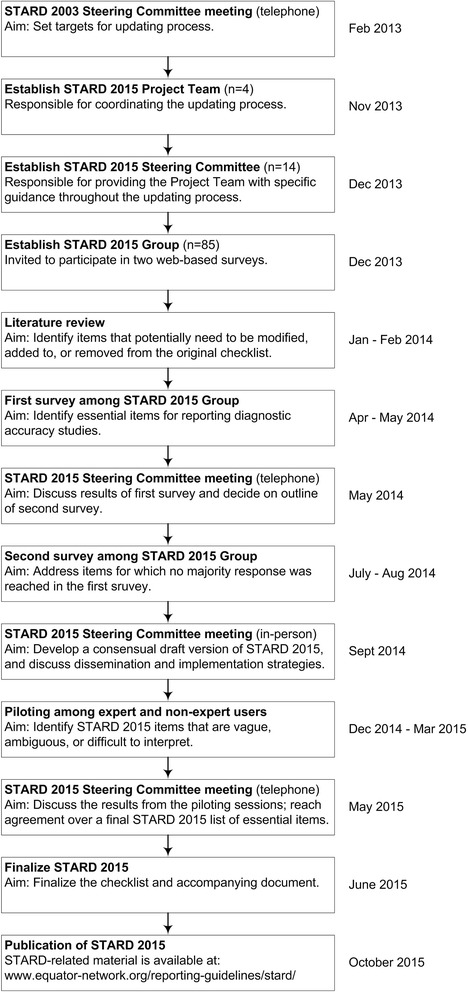
Milestones in the development of STARD 2015

Figure 1 summarizes our approach for updating STARD and lists critical milestones.

### Participants in the development of STARD 2015

The following groups of participants, detailed in Additional file 1, were involved in the STARD updating process.

#### Project Team

A four-member STARD 2015 Project Team was established, which was responsible for coordinating the updating process. This team secured funding, identified and invited potential new members of the STARD Group, reviewed the literature, conducted and analyzed web-based surveys, organized an in-person consensus meeting, drafted the items and accompanying documents, and coordinated piloting of the resulting STARD 2015 list.

#### Steering Committee

A 14-member STARD 2015 Steering Committee was also established, which was responsible for providing the Project Team with specific guidance throughout the updating process. This committee consisted of all ten members of the STARD 2003 Steering Committee [14], along with three journal editors from *Clinical Chemistry*, *JAMA*, and *Radiology*, and the founder of the EQUATOR Network (Enhancing the Quality and Transparency of Health Research), an umbrella organization that promotes complete and transparent reporting [15].

#### STARD Group

All 30 members of the original STARD 2003 Group were invited to contribute to the updating process and to suggest potential new members. Other potential new STARD Group members were identified from STARD-related publications during discussions within the Project Team. The resulting STARD 2015 Group now has 85 members; it consists of researchers, journal editors, healthcare professionals, methodologists, a science journalist, statisticians, other stakeholders, and the members of the Project Team and Steering Committee. STARD Group members were invited to participate in two web-based surveys to help identify essential items for reporting diagnostic accuracy studies.

### Review of the literature

In January and February 2014, the Project Team undertook a review of the literature to identify items that could be modified, added to, or removed from the original STARD checklist. This literature search focused on eight areas, which are detailed in Additional file 2.

In short, we searched MEDLINE (through PubMed) and the Cochrane Methodology Register, supplemented by non-systematic searches, for topics and items potentially relevant to the STARD updating process in three categories: (1) general considerations about diagnostic accuracy studies and reporting, (2) evidence and statements suggesting modifications to the original STARD checklist or flow diagram, and (3) evidence and statements suggesting new STARD items.

Titles and abstracts were screened by one of two reviewers (DAK or JFC), and potentially eligible publications were retrieved for full-text assessment, again by one of these two reviewers. The electronic search results were augmented by the personal article collections of the Project Team. Based on the results of this search, the Project Team decided which items should be presented for consideration to the STARD Group in an online survey.

### Online survey

#### General structure

We used two web-based surveys to help decide on items that needed to be modified, added to, or removed from the STARD checklist [12]. The surveys were developed by the Project Team in SurveyMonkey© and informally piloted in their institution prior to distribution.

All 85 members of the STARD Group were invited by email to participate in each survey. Near the closing dates, non-responders were sent two reminders, 1 week apart.

Participant responses were summarized by the Project Team and reported back to participants at the end of each survey. The Project Team and Steering Committee had a teleconference in May 2014 to discuss the results of the first survey and to decide on the outline of the second survey. They also set priorities for topics to discuss during the in-person consensus meeting.

#### First survey

A link to the first survey was sent to the STARD Group on April 16, 2014; the survey was closed on May 31, 2014. The questionnaire consisted of two parts, each containing a set of multiple-choice questions and is provided in Additional file 3.

In the first part of the questionnaire, participants were asked to comment on each of the 25 original STARD items, in order of their appearance in the original checklist. For each item, participants were invited to indicate whether they would prefer to keep the item as it is, to modify the item, or remove the item from the checklist. Each question was accompanied by a suggestion from the Project Team, supported by a brief rationale, based on the literature search results. Each question also contained an open-comment box in which participants could clarify their responses.

In the second part of the questionnaire, participants were asked whether or not they felt that proposed potential new items should be added to the list. The questionnaire also addressed general considerations about the scope of STARD and preferred wording and a box for further suggestions.

#### Second survey

A link to the second survey was distributed to the STARD Group on July 16, 2014; this survey closed on August 30, 2014. The invitation letter contained a document that summarized the results of the first survey. The questionnaire is provided in Additional file 4.

This second survey focused on items for which less than 75 % of the responders agreed on one of the multiple choice options in the first survey. Response options that had been selected by less than 20 % of the respondents in the first survey were removed from the questionnaire. Based on the open comments provided by the respondents in the first survey, a brief summary of the main arguments for and against each proposed modification was presented for each item.

Results from the second survey were summarized by the Project Team and used to prepare the first draft version of STARD 2015. Items for which there was no majority response were considered high-priority topics for discussion during the in-person consensus meeting.

### In-person consensus meeting

The 14 members from the STARD 2015 Steering Committee were invited to a 2-day consensus meeting, held in Amsterdam, The Netherlands, on September 27–28, 2014. The meeting was organized, coordinated, and chaired by the Project Team. The primary objective was to develop a consensual draft version of STARD 2015. Secondary objectives were to discuss dissemination and implementation plans for STARD 2015 and additional initiatives around STARD and to discuss how STARD 2015 could be integrated into long-term development strategies of the EQUATOR network [15].

After the meeting, Project Team members further revised the consensual draft version of STARD 2015, with collected comments and suggestions, and modified the prototype flow diagram that was provided in the original STARD statement. The updated consensual draft version was circulated by email to the STARD Group for feedback. The Project Team collected comments and suggestions and modified the list accordingly.

### Piloting STARD 2015

Three rounds of piloting among expert and non-expert users of STARD were organized. The main aim of these piloting sessions was to identify items on the consensual draft version of STARD 2015 that were vague, ambiguous, difficult to interpret, or missing.

#### Piloting among radiology residents

STARD 2015 was piloted among radiology residents from the Department of Radiology, Academic Medical Center, University of Amsterdam, The Netherlands. Residents were invited through email to read a diagnostic accuracy study report [16] and to use the checklist to evaluate completeness of reporting. This was followed by a focus group meeting, which took place on December 15, 2014. During a 90-min conversation, the moderator (DAK) invited the participants to comment on the wording and on the layout of the list. Thereafter, participants were invited to share how they had evaluated each item in the article provided and their experience with using the checklist.

#### Piloting among radiology experts

The editor-in-chief of *Radiology* invited editorial board members and reviewers of diagnostic accuracy studies to pilot the consensual draft version of STARD 2015 and to provide comments using an online questionnaire developed by the Project Team (Additional file 5). Responses were collected in SurveyMonkey© between January 9 and April 1, 2015. Invitees were asked to answer eight “yes/no/no opinion” questions about the list, with the option to clarify answers in an open-comment box. Specifically, they were asked whether the aim of STARD 2015 was clear; whether terminology, layout, and outline used were appropriate; and whether any item or information was particularly difficult to understand or missing.

#### Piloting among laboratory medicine experts

The editor-in-chief of *Clinical Chemistry* invited editors and reviewers of the journal to evaluate the consensual draft version of STARD 2015. Responses were collected between February 26 and March 9, 2015. Collaborators were asked to review the list and to provide feedback on whether they found the language understandable and the items sufficiently clear. They were also asked to indicate if any information deemed essential in evaluating laboratory medicine diagnostic accuracy studies was currently not addressed. This was done by email.

### Finalizing STARD 2015

The consensual draft version of the STARD 2015 list was updated following the piloting sessions. The Project Team summarized the feedback obtained from piloting and shared the results with the Steering Committee. In a teleconference on May 7, 2015, the Project Team and the Steering Committee decided on the final STARD 2015 list of essential items.

### Initial strategies for disseminating STARD 2015

In August 2015, we non-systematically searched PubMed for editorials and news items that had been published about STARD since its launch in 2003, and 33 were identified, published in 28 different journals. One author (JFC) collected the email addresses of the editors-in-chief or the editorial offices of these publishing journals. On November 26, 2015, these were contacted to inform them about the STARD 2015 update and to invite them to write an editorial or commentary around it.

In August 2015, we also searched PubMed for diagnostic accuracy studies that had been published between January and December 2014, using the following strategy: (sensitivity[tw] AND specificity[tw]) OR diagnostic accuracy[tw] OR predictive value*[tw] OR likelihood ratio*[tw] OR AUC[tw] OR ROC[tw]). We then ordered the search results by journal and established a list of the 100 journals that published most studies. For these journals, one author (DAK) collected the email addresses of the editors-in-chief or the editorial offices, and these were contacted on February 4, 2016, to inform them about the STARD 2015 update, and with the request to consider using and endorsing it.

## Results

### Review of the literature

A total of 113 full-text articles and reports were reviewed in preparation for the STARD 2015 update. A summary of the results of the literature review is provided in Additional file 6.

Based on the results of this review process, the Project Team decided to present 33 items—the 25 original items and 8 new items—for consideration to the STARD Group in the online survey. These eight potential new items were (1) positivity cutoffs for continuous tests when reporting area under the receiver operating characteristic curve, (2) sample size calculation, (3) trial registration number, (4) link to online resources, (5) availability of the study protocol, (6) data sharing policy, (7) conflicts of interest, and (8) sources of funding.

### Online survey

#### First survey

Seventy-three STARD Group members (86 %) completed the first survey. Detailed survey results are provided in Additional file 7. For the 25 items in the original STARD checklist, more than three quarters of respondents agreed to keep five items as they were (original STARD items 5/10/17/18/21) and to modify 13 items (original STARD items 2/4/6/8/9/11/12/13/14/16/19/22/24). There was less than 75 % agreement on the seven other items (original STARD items 1/3/7/15/20/23/25). Of the eight potential new items proposed, more than 75 % of respondents voted in favor of including four: sample-size calculation, availability of the study protocol, conflicts of interest, and sources of funding.

#### Second survey

Seventy-nine STARD Group members (93 %) completed the second survey. Detailed survey results are provided in Additional file 7. The survey addressed eight remaining questions: six items on the original STARD checklist for which less than 75 % of respondents indicated the same answer in the first survey (original STARD items 3/7/15/20/23/25), one potential new item (positivity cutoffs for continuous tests when reporting area under the receiver operating characteristic curve), and one wording issue (continuing to use the term “diagnostic accuracy” rather than moving to “diagnostic performance” as the key concept in reporting comparisons of medical tests with a clinical reference standard). More than 75 % voted to keep original STARD item 20 unchanged and to modify item 23 as suggested by the Project Team. No majority response was obtained for the other six questions.

### In-person consensus meeting

The Project Team and all but 3 of the 14 members of the Steering Committee attended the in-person consensus meeting (Additional file 1). On the first day, the items in the draft version of STARD 2015 and items for which no 75 % majority response were reached in the survey were discussed until consensus was reached on inclusion and phrasing. Thereafter, discussions focused on dissemination and uptake by journals, research institutions and authors, and strategies for piloting the list. It was also decided that a subgroup should develop a one-page explanatory document that briefly describes the aims of STARD 2015 and the key concepts in it to accompany the 2015 version when distributed.

On the second day, further discussions focused on finalizing a consensual draft version of STARD 2015. After this, additional initiatives around STARD were discussed. The meeting participants agreed that it would be valuable to develop extensions of STARD with more specific guidance for reporting diagnostic accuracy studies in different research fields (e.g., laboratory medicine and radiology) and applications of STARD for specific forms of testing (e.g., physical examination) or specific target conditions (e.g., dementia). The group agreed that STARD should also develop guidance for writing abstracts of diagnostic accuracy studies (STARD for Abstracts; in progress) and for registering protocols of diagnostic accuracy studies in trial registries (STARD for Registration; in progress).

### Piloting STARD 2015

#### Piloting among radiology residents

Four radiology residents (three men, one woman; age range 25–35 years; two of them with a PhD) participated in the initial piloting. Three of them declared being aware of the existence of STARD; two had previously used STARD for the critical appraisal of a diagnostic accuracy report they had to present during weekly journal clubs at the Department of Radiology. Comments of the participants were collected. From the interviews, we concluded that a majority of items on the consensual draft version of STARD 2015 were relevant and understandable by non-expert users. Residents suggested minor rewording for some items, adding explanation of key terms (such as “target condition” and “intended use of a test”), and a pointer to STARD for Abstracts currently in development.

#### Piloting among radiology experts

Twenty editorial board members and peer reviewers from *Radiology* completed the online piloting survey. Seventeen respondents were clinical radiologists, 2 were journal editors, and 1 was a biomedical researcher. All but one respondent declared having previously (co-)authored a diagnostic accuracy study. Detailed results are provided in Additional file 8. Most respondents considered the consensual draft version of the STARD 2015 list of essential items and accompanying one-page explanatory document as understandable and complete.

#### Piloting among laboratory medicine experts

Eight experts in the field of laboratory medicine provided feedback on the consensual draft version of STARD 2015 and the one-page explanation. Three experts indicated that the current draft version may not cover important elements of laboratory test evaluations, such as reproducibility of tests and collection, handling, and storage of samples. These experts highlighted the need for specific extensions or complementary documents dedicated to laboratory tests. Some respondents also suggested minor modifications and edits to the list.

### Finalizing STARD 2015

Amended draft versions of STARD 2015 were prepared. Based on the feedback provided during piloting, a new item pointing to STARD for Abstracts was added to the checklist, and a table to clarify key STARD terminology was developed [13]. Additional changes at this stage consisted mostly of minor wording modifications. On May 7, 2015, the Project Team and Steering Committee met in a teleconference during which the results from the piloting sessions were discussed, and STARD 2015 was finalized (Table 1) [13].

**Table 1:** The STARD 2015 list

Section and topic	No.	Item
Title or abstract		
	1	Identification as a study of diagnostic accuracy using at least one measure of accuracy (such as sensitivity, specificity, predictive values, or AUC)
Abstract		
	2	Structured summary of study design, methods, results, and conclusions (for specific guidance, see STARD for Abstracts)
Introduction		
	3	Scientific and clinical background, including the intended use and clinical role of the index test
	4	Study objectives and hypotheses
Methods		
Study design	5	Whether data collection was planned before the index test and reference standard were performed (prospective study) or after (retrospective study)
Participants	6	Eligibility criteria
	7	On what basis potentially eligible participants were identified (such as symptoms, results from previous tests, inclusion in registry)
	8	Where and when potentially eligible participants were identified (setting, location, and dates)
	9	Whether participants formed a consecutive, random, or convenience series
Test methods	10a	Index test, in sufficient detail to allow replication
	10b	Reference standard, in sufficient detail to allow replication
	11	Rationale for choosing the reference standard (if alternatives exist)
	12a	Definition of and rationale for test positivity cutoffs or result categories of the index test, distinguishing pre-specified from exploratory
	12b	Definition of and rationale for test positivity cutoffs or result categories of the reference standard, distinguishing pre-specified from exploratory
	13a	Whether clinical information and reference standard results were available to the performers or readers of the index test
	13b	Whether clinical information and index test results were available to the assessors of the reference standard
Analysis	14	Methods for estimating or comparing measures of diagnostic accuracy
	15	How indeterminate index test or reference standard results were handled
	16	How missing data on the index test and reference standard were handled
	17	Any analyses of variability in diagnostic accuracy, distinguishing pre-specified from exploratory
	18	Intended sample size and how it was determined
Results		
Participants	19	Flow of participants, using a diagram
	20	Baseline demographic and clinical characteristics of participants
	21a	Distribution of severity of disease in those with the target condition
	21b	Distribution of alternative diagnoses in those without the target condition
	22	Time interval and any clinical interventions between index test and reference standard
Test results	23	Cross tabulation of the index test results (or their distribution) by the results of the reference standard
	24	Estimates of diagnostic accuracy and their precision (such as 95 % confidence intervals)
	25	Any adverse events from performing the index test or the reference standard
Discussion		
	26	Study limitations, including sources of potential bias, statistical uncertainty, and generalizability
	27	Implications for practice, including the intended use and clinical role of the index test
Other information		
	28	Registration number and name of registry
	29	Where the full study protocol can be accessed
	30	Sources of funding and other support; role of funders

**Fig. 2:**
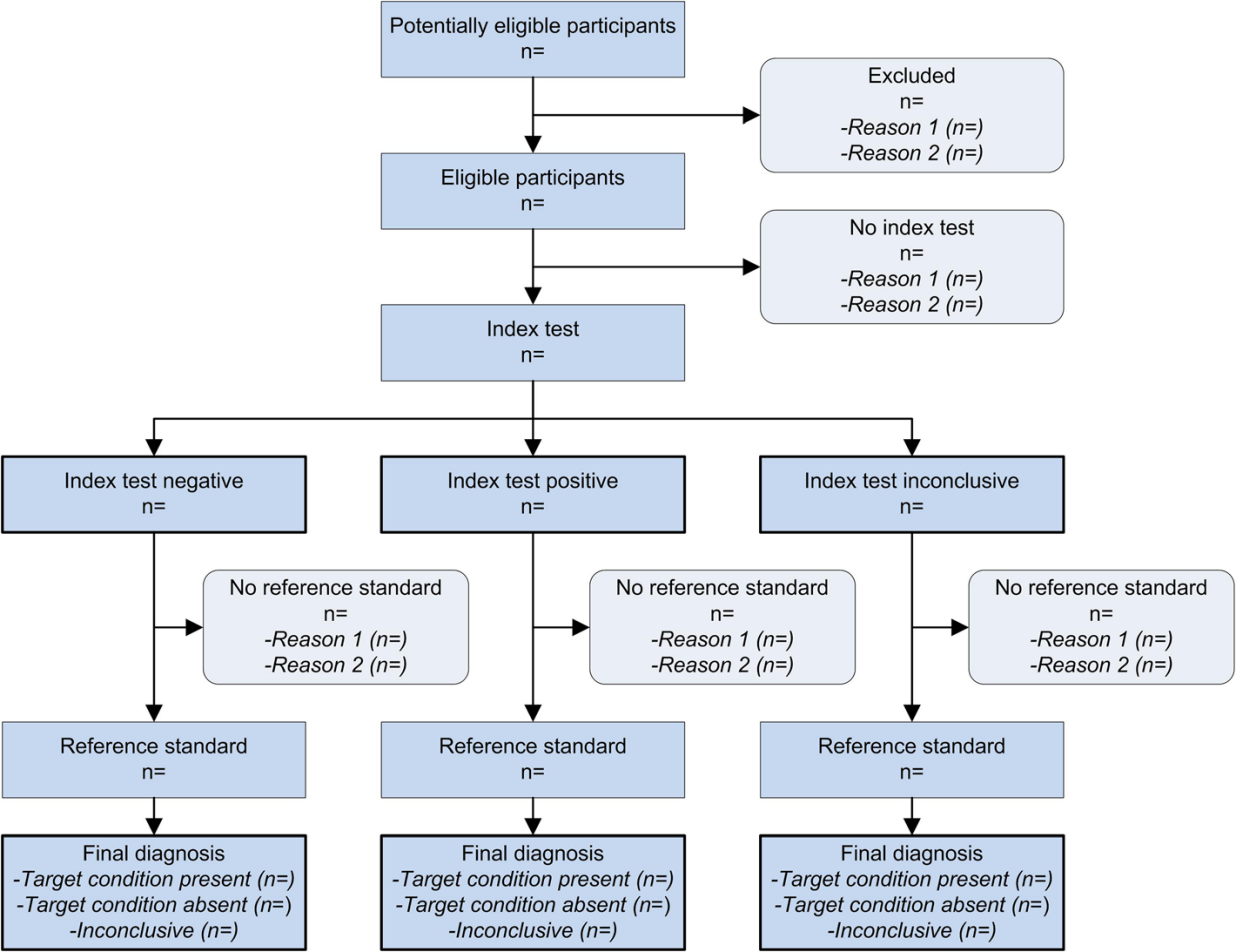
Prototypical STARD diagram to report flow of participants through the study

STARD 2015 consists of 30 items, with 4 items having an (a) and (b) part. A detailed rationale for modifying or adding items is provided in Additional file 9, with a summary of the main changes in Table 2. Compared to the original STARD checklist, three original items were each converted into two new items, four original items were incorporated into other items and seven completely new items were added. A modified prototype flow diagram, to illustrate the flow of participants through the study, was incorporated (Fig. 2). The remaining items were reworded to make them easier to understand or to bring them in line with phrasing used in other major reporting guidelines, such as CONSORT. [11] STARD 2015 now also has an accompanying one-page explanatory document that can be distributed along with it (Additional file 10). An updated “Explanation and Elaboration” document, which explains each item in detail and gives examples of good reporting [2], is under development; this document will be submitted for publication.

**Table 2:** Summary of main changes in STARD 2015

Section	Authors are invited to..
Title/abstract	report a structured abstract, according to STARD for Abstracts (item 2).
Introduction	report the intended use and clinical role of the index test under investigations (item 3), along with specific study hypotheses (item 4).
Methods	report whether test positivity cutoffs or result categories were pre-specified or exploratory (item 12), whether analyses of variability in diagnostic accuracy were pre-specified or exploratory (item 17), and how they determined the intended sample size (item 18).
Results	always provide a diagram, illustrating the flow of participants through the study (item 19).
Discussion	discuss potential study limitations (item 26) and the implications for practice of the study findings (item 27).
Other information	report the registration number (item 28), where the full study protocol can be accessed (item 29), and sources of funding (item 30).

A detailed overview of the changes made in STARD 2015, and the rationale for these changes, is provided in Additional file 9

The STARD 2015 list and the explanatory document have been released under a Creative Commons license that allows for redistribution, commercial and non-commercial use, as long as it is passed along unchanged and in whole, with credit to the STARD Group. All STARD-related material will be made accessible through the EQUATOR website upon completion (www.equator-network.org/reporting-guidelines/stard/).

## Discussion

Having completed the update of STARD, we would like to share a few observations and reflections. These can be read as limitations that we acknowledge, encouragement for others who are considering an update or an extension of a reporting guideline, and background information for users of reporting guidelines, such as STARD.

Even though STARD intends to cover reports of all studies that provide estimates of a test’s diagnostic accuracy, it may not be adequate to serve the special needs of each field. For specific types of tests and specific applications of testing, readers may wish to have more information to help them interpret and appreciate the study findings. The STARD Group encourages the development of extensions of STARD specifically designed for different fields of diagnostic research, and development of STARD applications, explaining how the STARD items should be operationalized for specific forms of testing or target conditions [17, 18]. Such extensions should not replace the whole of STARD, but rather modify or extend individual items, or possibly just interpret the items in a particular context. More details on how to develop extensions have been reported elsewhere [13].

Based on the accumulated experience since the development of STARD in 2003, we now firmly believe that developing a reporting checklist is in itself not sufficient to improve reporting [19]. We now see STARD 2015 as a list of essential items that provides a basis from which additional instruments have to be developed to address the needs of particular audiences. Though based on the STARD 2015 items, these instruments may differ, as they will target different potential users: not only authors of completed studies but also peer reviewers, journal editors, authors of conference abstracts, authors of study protocols, maybe even readers. Such instruments could, for example, be specific templates with standard text for authors, to facilitate complete reporting, or prototype statements for peer reviewers, who can point to reporting failures and explain why they need to be addressed. A writing aid for authors has been shown to be beneficial for improving reporting of randomized trials [20]. Other instruments that can be derived from the STARD 2015 items are guidance for reporting journal and conference abstracts and for registration of protocols of diagnostic accuracy studies in trial registries, initiatives that are currently ongoing.

Most reporting guidelines have not undergone user testing prior to their release, which may be surprising, given that reporting guidelines are primarily tools designed to help others, and they should be evaluated as such. We therefore decided to pilot STARD 2015 among different groups of potential users. This piloting was still relatively modest, but it helped us to improve the list in several key respects, especially in terms of wording.

Although we substantially extended membership of the STARD Group, the STARD 2015 update process mostly included experienced researchers and authors, and most of them were from USA, UK, or The Netherlands. To judge the formulation and user friendliness of items, the opinion of future users is important as well. The selection of items should be based on strong evidence and sound principles but the development of actual tools and instruments should be guided by repeated, targeted, and methodical user testing.

## Conclusion

After a systematic updating process, STARD 2015 provides an updated list of 30 essential items for reporting diagnostic accuracy studies. Incomplete reporting is now considered to be one of the largest sources of avoidable waste in biomedical research [21]. We believe that reporting can be substantially improved, with relatively little effort from multiple parties: from those responsible for training researchers, from the authors themselves, from journal editors, from peer reviewers, and from funders [22]. We invite all stakeholders to help disseminate STARD 2015 and to help the STARD Group in its efforts to promote more complete, more transparent, and more informative reporting of evaluations of medical tests.

## Additional files

Additional file 1: The STARD 2015 Group. (DOCX 38 kb)

Additional file 2: Literature review: search strategy. (DOCX 35 kb)

Additional file 3: First online questionnaire. (PDF 502 kb)

Additional file 4: Second online questionnaire. (PDF 208 kb)

Additional file 5: Questionnaire for piloting among experts in the field of imaging. (PDF 83 kb)

Additional file 6: Literature review: results. (DOCX 141 kb)

Additional file 7: Summary of responses to the online surveys. (DOCX 31 kb)

Additional file 8: Feedback from editorial board members and reviewers from *Radiology*. (DOCX 28 kb)

Additional file 9: Rationale for STARD 2015 items. (DOCX 45 kb)

Additional file 10: One-page explanation of STARD 2015. (DOCX 32 kb)

## Abbreviations

CONSORT: Consolidated Standards of Reporting Trials
EQUATOR: Enhancing the Quality and Transparency of Health Research
STARD: Standards for Reporting Diagnostic Accuracy
