# Supplementary material for: Updating standards for reporting diagnostic accuracy: the development of STARD 2015
Source: Res Integr Peer Rev. 2016 Jun 7;1:7. doi: 10.1186/s41073-016-0014-7 (PMC5803584; doi:10.1186/s41073-016-0014-7)
Supplement: Supplementary file 1 — The STARD 2015 Group. (DOCX 38 kb) [file 41073_2016_14_MOESM1_ESM.docx]

**Additional file 1. The STARD 2015 Group**

| **Name** | **Affiliation** | **PT** | **SC** | **LM** |
| --- | --- | --- | --- | --- |
| Todd Alonzo | Department of Biostatistics, University of Southern California, Los Angeles, USA |  |  |  |
| Doug Altman | Centre for Statistics in Medicine, Nuffield Department of Orthopaedics, Rheumatology and Musculoskeletal Sciences, University of Oxford, Oxford, UK |  | **X** | **X** |
| Augusto Azuara-Blanco | Queen's University Belfast, UK |  |  |  |
| Lucas Bachmann | Medignition Inc. Research Consultants, Zurich, Switzerland |  |  |  |
| Jeffrey Blume | Department of Biostatistics, Vanderbilt University School of Medicine, Nashville, USA |  |  |  |
| Patrick Bossuyt | Department of Clinical Epidemiology, Biostatistics and Bioinformatics, Academic Medical Center, University of Amsterdam, the Netherlands | **X** | **X** | **X** |
| Isabelle Boutron | Centre de Recherche Épidémiologie et Statistique Sorbonne Paris Cité (CRESS-UMR1153), Inserm, Université Paris Descartes, France |  |  |  |
| David Bruns | Department of Pathology, University of Virginia School of Medicine and Health System, Charlottesville, USA |  | **X** | **X** |
| Harry Büller | Academic Medical Center, University of Amsterdam, the Netherlands |  |  |  |
| Frank Buntinx | Department of General Practice, KU Leuven, Belgium; University of Maastricht, the Netherlands |  |  |  |
| Sarah Byron | National Institute for Health and Care Excellence, London, United Kingdom |  |  |  |
| Stephanie Chang | Agency for Healthcare Research and Quality, Rockville, USA |  |  |  |
| Jérémie Cohen | Department of Clinical Epidemiology, Biostatistics and Bioinformatics, Academic Medical Center, University of Amsterdam, the Netherlands; Inserm UMR 1153 and Department of Paediatrics, Necker Hospital, AP-HP, Paris Descartes University, France | **X** |  | **X** |
| Richelle Cooper | UCLA Emergency Medicine Center, Los Angeles, USA |  |  |  |
| Jon Deeks | Department of Public Health, Epidemiology and Biostatistics, University of Birmingham, Edgbaston Campus, UK |  |  |  |
| Joris de Groot | Julius Center for Health Sciences and Primary Care, University Medical Center, University of Utrecht, the Netherlands |  |  |  |
| Nandini Dendukuri | Department of Epidemiology, Biostatistics and Occupational Health, McGill University, Montréal Canada |  |  |  |
| Riekie de Vet | Department of Epidemiology & Biostatistics, VU University Medical Center, Amsterdam, the Netherlands |  | **X** | **X** |
| Jac Dinnes | University of Birmingham, UK |  |  |  |
| Kenneth Fleming | Green Templeton College, University of Oxford, UK |  |  |  |
| Constantine Gatsonis | Center for Statistical Sciences, Brown University School of Public Health, Providence, Rhode Island, USA |  | **X** | **X** |
| Paul Glasziou | Centre for Research in Evidence-Based Practice, Bond University, Australia |  | **X** | **X** |
| Robert Golub | Deputy Editor, *JAMA*, USA |  | **X** | **X** |
| Gordon Guyatt | Clinical Epidemiology and Biostatistics, McMaster University, Hamilton, Canada |  |  |  |
| Carl Heneghan | Centre for Evidence-Based Medicine, Nuffield Department of Primary Care Health Sciences, University of Oxford, UK |  |  |  |
| Jorgen Hilden | University of Copenhagen, Copenhagen, Denmark |  |  |  |
| Lotty Hooft | Dutch Cochrane Centre, Julius Center for Health Sciences and Primary Care, University Medical Center, University of Utrecht, the Netherlands | **X** |  | **X** |
| Rita Horvath | SEALS Department of Clinical Chemistry, Prince of Wales Hospital; Screening and Test Evaluation Program, School of Public Health, University of Sydney; and School of Medical Sciences, University of New South Wales, Sydney, Australia |  |  |  |
| Myriam Hunink | Department of Epidemiology and Radiology, Erasmus MC, Rotterdam, the Netherlands; Centre for Health Decision Science, Harvard T.H. Chan School of Public Health, Boston, USA |  |  |  |
| Chris Hyde | University of Exeter Medical School, UK |  |  |  |
| John Ioannidis | Department of Medicine, Stanford Prevention Research Center, Stanford University Stanford, USA |  |  |  |
| Les Irwig | Screening and Test Evaluation Program, School of Public Health, University of Sydney, Australia |  | **X** |  |
| Holly Janes | Fred Hutchinson Cancer Research Center and University of Washington, Seattle, USA |  |  |  |
| Jos Kleijnen | School for Public Health and Primary Care (CAPHRI), Maastricht University, the Netherlands |  |  |  |
| Andre Knottnerus | Department of Family Medicine, School for Public Health and Primary Care (CAPHRI), Maastricht University, the Netherlands |  |  |  |
| Daniel Korevaar | Department of Clinical Epidemiology, Biostatistics and Bioinformatics, Academic Medical Center, University of Amsterdam, the Netherlands | **X** |  | **X** |
| Herbert Kressel | Miriam H. Stoneman Professor of Radiology, Harvard Medical School, Boston, USA; Editor, *Radiology*, Boston, USA |  | **X** | **X** |
| Stefan Lange | Institute for Quality and Efficiency in Health Care (IQWiG), Cologne, Germany |  |  |  |
| Mariska Leeflang | Department of Clinical Epidemiology, Biostatistics and Bioinformatics, Academic Medical Center, University of Amsterdam, the Netherlands |  |  |  |
| Jeroen Lijmer | OLVG Hospital, Amsterdam, the Netherlands |  | **X** | **X** |
| Sally Lord | School of Medicine, University of Notre Dame and NHMRC Clinical Trials Centre, University of Sydney, Australia |  |  |  |
| Blanca Lumbreras | Public Health Department, University Miguel Hernandez and CIBERESP (CIBER en Epidemiología y Salud Pública), Spain |  |  |  |
| Petra Macaskill | Screening and Test Evaluation Program, School of Public Health, University of Sydney, Australia |  |  |  |
| Erik Magid | Department of Clinical Biochemistry, Amager Hospital, Copenhagen, Denmark |  |  |  |
| Susan Mallett | Nuffield Department of Primary Care Health Sciences, University of Oxford, UK |  |  |  |
| Barbara McNeil | Department of Health Care Policy, Harvard Medical School, Boston, USA |  |  |  |
| Matthew McInnes | University of Ottawa Department of Radiology; Ottawa Hospital Research Institute Clinical Epidemiology Program, Canada |  |  |  |
| Matthew McQueen | McMaster University and the Population Health Research Institute, Hamilton, Canada |  |  |  |
| David Moher | Ottawa Hospital Research Institute; School of Epidemiology, Public Health and Preventive Medicine, Faculty of Medicine, University of Ottawa, Canada |  | **X** |  |
| Carl Moons | Julius Center for Health Sciences and Primary Care, University Medical Center, University of Utrecht, the Netherlands |  |  |  |
| Katie Morris | Birmingham Centre for Women and Children's Health, College of Medical and Dental Sciences, University of Birmingham, UK |  |  |  |
| Reem Mustafa | Department of Medicine/Nephrology and Biomedical & Health Informatics, University of Missouri-Kansas City School of Medicine, USA |  |  |  |
| Nancy Obuchowski | Cleveland Clinic Foundation, USA |  |  |  |
| Eleanor Ochodo | Centre for Evidence-based Health Care, Faculty of Medicine and Health Sciences, Stellenbosch University, Cape Town, South Africa |  |  |  |
| Andrew Onderdonk | Department of Pathology, Brigham and Women's Hospital, Harvard Medical School, Boston, USA |  |  |  |
| John Overbeke | Department of Primary and Community Care, Radboud University Medical Centre, Nijmegen, The Netherlands |  |  |  |
| Nitika Pai | Division of Clinical Epidemiology, Department of Medicine, McGill University,, Montreal, Canada |  |  |  |
| Rosanna Peeling | London School of Hygiene and Tropical Medicine, UK |  |  |  |
| Margaret Pepe | Fred Hutchinson Cancer Research Center, University of Washington, Seattle, USA |  |  |  |
| Steffen Petersen | William Harvey Research Institute, Queen Mary University of London, UK |  |  |  |
| Christopher Price | Department of Primary Care Health Sciences, University of Oxford, UK |  |  |  |
| Philippe Ravaud | Centre de Recherche Épidémiologie et Statistique Sorbonne Paris Cité (CRESS-UMR1153), Inserm, Université Paris Descartes, France |  |  |  |
| Hans Reitsma | Julius Center for Health Sciences and Primary Care, University Medical Center, University of Utrecht, the Netherlands |  | **X** | **X** |
| Drummond Rennie | Peer Review Congress, Chicago, Illinois, USA; Philip R. Lee Institute for Health Policy Studies, University of California, San Francisco, California, USA |  | **X** |  |
| Nader Rifai | Boston Children's Hospital and Harvard Medical School, Boston, USA |  | **X** | **X** |
| Anne Rutjes | CTU Bern, Department of Clinical Research, University of Bern, Switzerland; Centre for Systematic Reviews at Fondazione "Università G. D'Annunzio", Chieti, Italy |  |  |  |
| Holger Schunemann | Department of Clinical Epidemiology and Biostatistics, McMaster University, Hamilton, Canada |  |  |  |
| David Simel | Department of Medicine, Durham Veterans Affairs Medical Center, Duke University Health System, USA |  |  |  |
| Iveta Simera | UK EQUATOR Centre, University of Oxford, UK |  |  |  |
| Nynke Smidt | Department of Epidemiology, University Medical Center Groningen, the Netherlands |  |  |  |
| Ewout Steyerberg | Centre for Medical Decision Making, Department of Public Health, Erasmus MC-University Medical Centre Rotterdam, the Netherlands |  |  |  |
| Sharon Straus | Li Ka Shing Knowledge Institute of St Michael's, University of Toronto, Canada |  |  |  |
| William Summerskill | *The Lancet*, London, UK |  |  |  |
| Yemisi Takwoingi | Public Health, Epidemiology and Biostatistics, University of Birmingham, UK |  |  |  |
| Matthew Thompson | Professor of Family Medicine, University of Washington, Seattle, USA |  |  |  |
| Ann van den Bruel | Nuffield Department of Primary Care Health Sciences, University of Oxford, UK |  |  |  |
| Hans van Maanen | Science journalist, the Netherlands |  |  |  |
| Andrew Vickers | Memorial Sloan Kettering Cancer Center, New York, USA |  |  |  |
| Gianni Virgili | Department of Translational Surgery and Medicine, University of Florence, Italy |  |  |  |
| Stephen Walter | Department of Clinical Epidemiology and Biostatistics, McMaster University, Hamilton, Canada |  |  |  |
| Wim Weber | *BMJ*, London, UK |  |  |  |
| Marie Westwood | Kleijnen Systematic Reviews Ltd, York, UK |  |  |  |
| Penny Whiting | School of Social and Community Medicine, University of Bristol, UK |  |  |  |
| Nancy Wilczynski | McMaster University, Hamilton, Canada |  |  |  |
| Andreas Ziegler | Institute of Medical Biometry and Statistics and Centre for Clinical Trials, University of Lübeck, Germany |  |  |  |

Abbreviations: PT = Project Team; SC = Steering Committee; LM = Live Meeting participants.
