# Supplementary material for: Updating standards for reporting diagnostic accuracy: the development of STARD 2015
Source: Res Integr Peer Rev. 2016 Jun 7;1:7. doi: 10.1186/s41073-016-0014-7 (PMC5803584; doi:10.1186/s41073-016-0014-7)
Supplement: Supplementary file 2 — Literature review: search strategy. (DOCX 35 kb) [file 41073_2016_14_MOESM2_ESM.docx]

**Additional file 2. Literature review: search strategy**

1. **Sources of bias and variation in diagnostic accuracy studies**

This part of the search covers the methodological literature on bias in, and lack of applicability of, results of studies on diagnostic accuracy published since 2000.

Search strategy: non-systematic, based on Whiting et al. Journal of Clinical Epidemiology 2013 [[1](#_ENREF_1)]

1. **Methods for the design, conduct and analysis of diagnostic accuracy studies**

This part of the search focuses on methodology issues in performing diagnostic accuracy studies.

Search strategy:

- Part 1: MEDLINE

((Research Design/standards* [mesh] OR Research Design/methods*[Mesh] OR Epidemiologic Research Design/methods*[Mesh] OR Epidemiologic Research Design/standards*[Mesh] OR Research Report/standards*[mesh] OR Medical Laboratory Science/standards*[mesh]) AND (Diagnostic Techniques and Procedures/standards*[mesh] OR Diagnostic Tests, Routine/methods*[mesh] OR Diagnostic Tests, Routine/standards*[mesh] OR Sensitivity and Specificity[Mesh] OR diagnos*[ti])) AND ("2000/01/01"[Date - Publication] : "3000"[Date - Publication])

- Part 2: Cochrane Methodology Register

| #1 | diagnostic:ti,ab,kw from 2000 to 2014, in Methods Studies (Word variations have been searched) |
| --- | --- |
| #2 | reviews:ti from 2000 to 2014, in Methods Studies (Word variations have been searched) |
| #3 | #1 not #2 |

1. **Existing lists of criteria for the reporting of diagnostic accuracy studies and extensions of STARD**

This part of the search focuses on existing standards or sets of criteria used in the evaluation of diagnostic tests.

Search strategy: non-systematic

1. **Reporting issues: spin, overinterpretation, optimism, selective reporting**

Search strategy: non-systematic, based on Ochodo et al. Radiology 2013 [[2](#_ENREF_2)]

1. **Evaluations of adherence to STARD**

This part of the search aimed to identify and evaluate studies evaluating adherence to STARD in the literature. Poorly reported items might reveal unclear or ambiguous items on the STARD checklist.

Search strategy: non-systematic, based on Korevaar et al. Evidence BasedMedicine 2013 [[3](#_ENREF_3)]

1. **Other EQUATOR guidelines**

Here we aim to review the content of other EQUATOR guidelines in order to identify potential general items that could be added to STARD. We also aim to improve consistency in the content and wording of STARD with EQUATOR guidelines.

Search strategy: manual, EQUATOR website

1. **“Grey literature”**

Search strategy: non-systematic

1. **Articles from dr. John Ioannidis’ team**

John Ioannidis has proven to be a researcher of great value to clinical epidemiology. His team was productive during the past ten years and we aimed to review his works in the field of diagnosis, prognosis and reporting.

Search strategy (in MEDLINE):

(Ioannidis J[Author]) AND ("2000"[Date - Publication] : "3000"[Date - Publication])
