# Supplementary material for: Updating standards for reporting diagnostic accuracy: the development of STARD 2015
Source: Res Integr Peer Rev. 2016 Jun 7;1:7. doi: 10.1186/s41073-016-0014-7 (PMC5803584; doi:10.1186/s41073-016-0014-7)
Supplement: Supplementary file 3 — First online questionnaire. (PDF 502 kb) [file 41073_2016_14_MOESM3_ESM.pdf]

Welcome to the first round of the STARD update survey.

The following 25 items are in the current STARD checklist, but may need revision.
The “consideration” summarizes our rationale for the change.
Whenever our research suggested a specific modification, we have listed this as “our suggestion”.

Your comments are always welcome and will be taken into account in the preparations for the second round of the survey.

This survey is not anonymous.

Please enter your first name and last name.

Item 1: Identify the article as a study of diagnostic accuracy (recommend MeSH heading 'sensitivity and specificity').

Consideration:
A wide variety of terms is used to announce studies of diagnostic accuracy; “diagnostic accuracy” itself is not frequently used.

Should we:

- Keep this item as it is
- Modify this item: provide more guidance on which terms to use in the title and abstract
- Modify this item otherwise (please explain)
- Remove this item *(our suggestion)*
- No opinion

Open comment box:

Item 2: State the research questions or study aims, such as estimating diagnostic accuracy or comparing accuracy between tests or across participant groups.

Consideration:

Many diagnostic accuracy studies report vague and very general study aims.

Should we:

- Keep this item as it is
- Modify this item: invite authors to report the purpose, clinical context, and clinical role of the test, when describing the study aims *(our suggestion)*
- Modify this item otherwise (please explain)
- Remove this item
- No opinion

Open comment box:

Item 3: Describe the study population: The inclusion and exclusion criteria, setting and locations where the data were collected.

Consideration:

Setting and locations are features of participant recruitment, which is covered by item 4 of the checklist.

Should we:

- Keep this item as it is
- Modify this item: move “setting and locations” from item 3 to item 4 (“participant recruitment”) *(our suggestion)*
- Modify this item otherwise (please explain)
- Remove this item
- No opinion

Open comment box:

Item 4: Describe participant recruitment: Was recruitment based on presenting symptoms, results from previous tests, or the fact that the participants had received the index tests or the reference standard?

Consideration:

Long and confusing wording.

Should we:

- Keep this item as it is
- Modify this item: reword and simplify *(our suggestion)*
- Modify this item otherwise (please explain)
- Remove this item
- No opinion

Open comment box:

Item 5: Describe participant sampling: Was the study population a consecutive series of participants defined by the selection criteria in items 3 and 4? If not, specify how participants were further selected.

Consideration:

We did not observe major issues with this item.

Should we:

- Keep this item as it is *(our suggestion)*
- Modify this item (please explain)
- Remove this item
- No opinion

Open comment box:

Item 6: Describe data collection: Was data collection planned before the index test and reference standard were performed (prospective study) or after (retrospective study)?

Consideration:
There is widespread variability in the interpretation of the labels “prospective” and “retrospective”.
It is relevant to know in which order question formulation, data collection and analysis took place.

Should we:

- Keep this item as it is
- Modify this item: reword and simplify *(our suggestion)*
- Modify this item otherwise (please explain)
- Remove this item
- No opinion

Open comment box:

Item 7: Describe the reference standard and its rationale.

Consideration:
The rationale for the reference standard is often not reported, and typically not provided in the methods section.

Should we:

- Keep this item as it is
- Modify this item: remove "and its rationale" *(our suggestion)*
- Modify this item otherwise (please explain)
- Remove this item
- No opinion

Open comment box:

Item 8: Describe technical specifications of material and methods involved including how and when measurements were taken, and/or cite references for index tests and reference standard.

Consideration:
Other reporting guidelines are much more specific on which items to report for technical specifications.
The relevance of technical information reported may differ between types of tests (e.g. imaging, laboratory, other).

Should we:

- Keep this item as it is
- Modify this item: refer to list of preferred descriptions for specific test types (to be developed) *(our suggestion)*
- Modify this item otherwise (please explain)
- Remove this item
- No opinion

Open comment box:

Item 9: Describe definition of and rationale for the units, cut-offs and/or categories of the results of the index tests and the reference standard.

Consideration:
This item is ambiguous – accuracy does not depend on the unit of measurement, but may change with the cut-offs and categories chosen to classify test results.

Should we:

- Keep this item as it is
- Modify this item: remove “units” and invite authors to report whether cut-offs and/or categories were pre-specified *(our suggestion)*
- Modify this item otherwise (please explain)
- Remove this item
- No opinion

Open comment box:

Item 10: Describe the number, training and expertise of the persons executing and reading the index tests and the reference standard.

Consideration:
We did not observe major issues with this item.

Should we:

- Keep this item as it is *(our suggestion)*
- Modify this item (please explain)
- Remove this item
- No opinion

Open comment box:

Item 11: Describe whether or not the readers of the index tests and reference standard were blind (masked) to the results of the other test and describe any other clinical information available to the readers.

Consideration:
There is widespread variability in the interpretation of the label "blind".
It is important to know what information is available to the readers of the tests.
This item contains both a negative statement ("blinding") and a positive statement ("clinical information available").

Should we:

- Keep this item as it is
- Modify this item: reword and simplify *(our suggestion)*
- Modify this item otherwise (please explain)
- Remove this item
- No opinion

Open comment box:

Item 12: Describe methods for calculating or comparing measures of diagnostic accuracy, and the statistical methods used to quantify uncertainty (e.g. 95% confidence intervals).

Consideration:
The nature of statistical methods to be reported seems unclear to many authors: methods for the accuracy statistics, or for the uncertainty, or both?

Should we:

- Keep this item as it is
- Modify this item: reword and simplify *(our suggestion)*
- Modify this item otherwise (please explain)
- Remove this item
- No opinion

Open comment box:

Item 13: Describe methods for calculating test reproducibility, if done.

Consideration:
The word “reproducibility” is ambiguous.
Estimating a test’s reproducibility is not an element of most diagnostic accuracy studies.
Many studies refer to other publications or to the manufacturer for information on test reproducibility.

Should we:

- Keep this item as it is
- Modify this item (please explain)
- Remove this item, and integrate in item 8 (“technical specifications of index test and reference standard”) *(our suggestion)*
- Remove this item
- No opinion

Open comment box:

Item 14: Report when study was done, including beginning and ending dates of recruitment.

Consideration:

This item is almost always reported in the methods section, rarely in the results section, and refers to participant recruitment (item 4).

Should we:

- Keep this item as it is
- Modify this item (please explain)
- Remove this item, and integrate in item 4 ("participant recruitment") *(our suggestion)*
- Remove this item
- No opinion

Open comment box:

Item 15: Report clinical and demographic characteristics of the study population (e.g. age, sex, spectrum of presenting symptoms, co-morbidity, current treatments, recruitment centers).

Consideration:

Depending on the type of test and target condition, there is a very large variety in suitable clinical and demographic characteristics reported in diagnostic accuracy studies.

Should we:

- Keep this item as it is
- Modify this item: simplify and remove proposed characteristics ("e.g. age, sex, spectrum of presenting symptoms, ...") *(our suggestion)*
- Modify this item otherwise (please explain)
- Remove this item
- No opinion

Open comment box:

Item 16: Report the number of participants satisfying the criteria for inclusion that did or did not undergo the index tests and/or the reference standard; describe why participants failed to receive either test (a flow diagram is strongly recommended).

Consideration:
This is a lengthy and complex item.
Flow diagrams were strongly recommended in STARD, but these are only used in a minority of studies.

Should we:

- Keep this item as it is
- Modify this item: reword and always require a flow diagram *(our suggestion)*
- Modify this item otherwise (please explain)
- Remove this item
- No opinion

Open comment box:

Item 17: Report time interval from the index tests to the reference standard, and any treatment administered between.

Consideration:
We did not observe major issues with this item.

Should we:

- Keep this item as it is *(our suggestion)*
- Modify this item (please explain)
- Remove this item
- No opinion

Open comment box:

Item 18: Report distribution of severity of disease (define criteria) in those with the target condition; other diagnoses in participants without the target condition.

Consideration:

We did not observe major issues with this item.

Should we:

- Keep this item as it is *(our suggestion)*
- Modify this item (please explain)
- Remove this item
- No opinion

Open comment box:

Item 19: Report a cross tabulation of the results of the index tests (including indeterminate and missing results) by the results of the reference standard; for continuous results, the distribution of the test results by the results of the reference standard.

Consideration:

Long and confusing wording.

Indeterminate and missing results are almost never reported in cross tabulations.

It is important to know how indeterminate results, missing responses and outliers were handled, but this is already discussed in item 22.

Should we:

- Keep this item as it is
- Modify this item: simplify and remove the terms "including indeterminate and missing results" *(our suggestion)*
- Modify this item otherwise (please explain)
- Remove this item
- No opinion

Open comment box:

Item 20: Report any adverse events from performing the index tests or the reference standard.

Consideration:
Adverse events are rarely reported in diagnostic accuracy studies; such studies typically lack the power and design to estimate adverse event rates.
Many tests do not have intrinsic adverse events.

Should we:

- Keep this item as it is
- Modify this item (please explain)
- Remove this item *(our suggestion)*
- No opinion

Open comment box:

Item 21: Report estimates of diagnostic accuracy and measures of statistical uncertainty (e.g. 95% confidence intervals).

Consideration:
We did not observe major issues with this item.

Should we:

- Keep this item as it is *(our suggestion)*
- Modify this item (please explain)
- Remove this item
- No opinion

Open comment box:

Item 22: Report how indeterminate results, missing responses and outliers of the index tests were handled.

Consideration:

Authors should be encouraged to plan ahead how to handle indeterminate results, missing responses and outliers in their study protocol.

Should we:

- Keep this item as it is
- Modify this item: move to the “methods” items *(our suggestion)*
- Modify this item otherwise (please explain)
- Remove this item
- No opinion

Open comment box:

Item 23: Report estimates of variability of diagnostic accuracy between subgroups of participants, readers or centers, if done.

Consideration:

Test accuracy may vary across subgroups but many diagnostic accuracy studies lack the power to detect such variations.

Variability is often not reported.

Multiple subgroup analyses can increase the risk of false-positive findings.

Should we:

- Keep this item as it is
- Modify this item: invite authors to report whether subgroup analyses were pre-planned *(our suggestion)*
- Modify this item otherwise (please explain)
- Remove this item
- No opinion

Open comment box:

Item 24: Report estimates of test reproducibility, if done.

Consideration:
The word “reproducibility” is ambiguous.
Estimating a test’s reproducibility is not an element of most diagnostic accuracy studies.
Many studies refer to other publications or to the manufacturer for information on test reproducibility.

Should we:

- Keep this item as it is
- Modify this item (please explain)
- Remove this item, and integrate in item 8 (“technical specifications of index test and reference standard”) *(our suggestion)*
- Remove this item
- No opinion

Open comment box:

Item 25: Discuss the clinical applicability of the study findings.

Consideration:
This item is rather vague, general and not specific for diagnostic accuracy studies.
Many reports of test accuracy studies offer generous and optimistic interpretations of the study findings, with strong recommendations for practice.

Should we:

- Keep this item as it is
- Modify this item (please explain)
- Remove this item
- No opinion

Open comment box:

Welcome to the first round of the STARD update survey.

The following items and issues were identified based on our literature review and comparisons between STARD and other reporting guidelines.

Your comments are always welcome and will be taken into account in the preparations for the second round of the survey.

This survey is not anonymous.

Please enter your first name and last name.

PROPOSALS FOR NEW ITEMS

Sample size

Consideration:
Sample size calculations are rarely reported in diagnostic accuracy studies.

Should STARD recommend reporting the method and rationale for the study sample size calculation?

- Yes
- No
- No opinion

Open comment box:

PROPOSALS FOR NEW ITEMS

Cut-offs for continuous tests

Consideration:
Many diagnostic accuracy studies report area under the receiver operator curve (AUC-ROC).
Without accuracy estimates at specific cut-offs, such a result is difficult to apply.

Should STARD recommend reporting at least one cut-off when reporting AUC-ROC?

- ○ Yes
- ○ No
- ○ No opinion

Open comment box:

PROPOSALS FOR NEW ITEMS

Additional information

Consideration:
There is a movement towards more openness and transparency in health research in general. This is not specific for test accuracy studies.

Should STARD recommend reporting...

	Yes	No	No opinion
...the trial registration number?	○	○	○
...a link to online resources with more information on the study?	○	○	○
...about the availability of the study protocol?	○	○	○
...about the availability of patient level data, or the data sharing policy?	○	○	○
...conflicts of interest?	○	○	○
...sources of funding?	○	○	○

Open comment box:

GENERAL CONSIDERATIONS

Scope of STARD

Consideration:
STARD was originally targeted at “diagnostic accuracy studies”.
Many other studies also report accuracy estimates, as an additional aim.

Should the applicability of STARD be rephrased, from “diagnostic accuracy studies” to “studies reporting diagnostic accuracy”?

- Yes
- No
- No opinion

Open comment box:

GENERAL CONSIDERATIONS

Scope of STARD

Consideration:
STARD was originally targeted at “diagnostic accuracy studies”, which are cross-sectional.
In practice, we also see diagnostic studies with so-called “delayed verification”, and other studies reporting on prognostic accuracy.

Should the applicability of STARD be extended to prognostic accuracy studies?

- Yes
- No
- No opinion

Open comment box:

GENERAL CONSIDERATIONS

Scope of STARD

Consideration:
Medical tests are not just used for diagnosis and prognosis, but also for other purposes, such as screening, monitoring or treatment selection.
Many, if not all, STARD items also apply to studies evaluating the accuracy of such tests.

Should the applicability of STARD be rephrased, from “diagnostic accuracy” to “(clinical) test accuracy”?

- Yes
- No
- No opinion

Open comment box:

GENERAL CONSIDERATIONS

Scope of STARD

Consideration:
The emphasis in STARD was on studies of a single (index) test, but the principles also apply to evaluations of the accuracy of multiple tests, combinations of tests, and multivariable models and rules.

Should the applicability of STARD be rephrased, e.g. in terms of “all evaluations of the accuracy of one or more tests, or combinations of test results and/or other variables”?

- Yes
- No
- No opinion

Open comment box:

GENERAL CONSIDERATIONS

Preferred wording

Consideration:
There is a wide variety of terms used to describe elements of a diagnostic accuracy study.

Should STARD recommend preferred terms for indicating...

	Yes	No	No opinion
...the type of study (e.g. “a diagnostic accuracy study” or “a test accuracy study”)?	○	○	○
...the study design (e.g. cohort/case-control or single-gate/multiple-gate studies)?	○	○	○
...the “index test” and the “clinical reference standard”?	○	○	○

Open comment box:
