# Supplementary material for: Updating standards for reporting diagnostic accuracy: the development of STARD 2015
Source: Res Integr Peer Rev. 2016 Jun 7;1:7. doi: 10.1186/s41073-016-0014-7 (PMC5803584; doi:10.1186/s41073-016-0014-7)
Supplement: Supplementary file 4 — Second online questionnaire. (PDF 208 kb) [file 41073_2016_14_MOESM4_ESM.pdf]

STARD 2014: Round 2

Welcome to the second STARD update survey.

In this survey we present STARD items for which there was no majority response about the suggested modifications. We also present a very brief summary of the main arguments for or against modifications. Response options that were not or rarely selected in the first survey have been removed.

Your comments are always welcome and will be taken into account in further preparing the STARD update.

This survey is also not anonymous.

Could you please enter your first and last name.

Item 3: Describe the study population: The inclusion and exclusion criteria, setting and locations where the data were collected.

Considerations:
Setting and locations are features of participant recruitment, which is covered by item 4 of the STARD checklist.
In the first round, we suggested to move “setting and locations” from item 3 to item 4, but some STARD group members argued that setting is an essential element of the inclusion criteria and should therefore be kept as part of item 3.

Should we:

- Keep item 3 as it is
- Reword item 3 into “Study population - Describe the inclusion and exclusion criteria” (“setting and locations” to be included in item 4, under “Describe participant recruitment”)
- No opinion

Open comment box:

Item 7: Describe the reference standard and its rationale.

Considerations:

The rationale for the reference standard is often not reported, and typically not provided in the methods section.

In the first round, we suggested to remove "and its rationale" but some argued that whenever there exists more than one reference standard for a target condition, the rationale for the reference standard selected in the study should be explained.

Should we:

- Keep item 7 as it is
- Modify item 7 into "Describe the reference standard"
- Modify item 7: reword to make clear that "rationale" refers to the choice of a reference standard among alternatives
- No opinion

Open comment box:

Item 15: Report clinical and demographic characteristics of the study population (e.g. age, sex, spectrum of presenting symptoms, co-morbidity, current treatments, recruitment centers).

Considerations:

There is a large variability in clinical and demographic characteristics that can be reported in diagnostic accuracy studies. Having shorter STARD items may be preferable.

In the first round, we proposed to remove the suggested characteristics but some argued that examples are needed, to illustrate what the item means.

Should we:

- Keep item 15 as it is
- Modify item 15 into "Report demographic and clinical characteristics of the study participants" and provide more guidance in the explanatory document
- No opinion

Open comment box:

Item 20: Report any adverse events from performing the index tests or the reference standard.

Considerations:
Many tests do not have intrinsic adverse events, but some do. Accuracy studies may be the only studies to report on a test. On the other hand, such studies typically lack the power for precise estimates of adverse event rates.
In the first round, we suggested to remove this item but some argued that this would reinforce the incorrect belief that adverse events are not important.

Should we:

- ○ Keep item 20 as it is
- ○ Remove item 20
- ○ No opinion

Open comment box:

Item 23: Report estimates of variability of diagnostic accuracy between subgroups of participants, readers or centers, if done.

Considerations:
Test accuracy may vary across subgroups but many diagnostic accuracy studies lack the power to detect such differences. Multiple subgroup analyses can increase the risk of false-positive findings.
In the first round, we suggested to ask authors to only report whether subgroup analyses were preplanned. Some STARD group members argued that STARD should also provide guidance on how to adequately report results of subgroup analyses.

Should we:

- ○ Modify item 23: invite authors to report in the methods whether subgroup analyses were preplanned
- ○ Modify item 23: invite authors to report in the methods whether subgroup analyses were preplanned and provide guidance on how to report subgroup differences in the explanatory document
- ○ No opinion

Open comment box:

Item 25: Discuss the clinical applicability of the study findings.

Considerations:
We did not have any suggestion about this item in the first round. Many respondents pointed to the need for a structured discussion addressing the internal validity, applicability and implications for practice and research, as for other reporting guidelines. On the other hand, a structured discussion is not a specific requirement for diagnostic accuracy studies and journals may have specific instructions for organizing the discussion section.

Should we:

- ○ Keep item 25 as it is
- ○ Modify item 25: invite authors to adopt a structured discussion
- ○ Remove this item
- ○ No opinion

Open comment box:

Potential new item: Cut-offs with AUC-ROC.

Considerations:
Many diagnostic accuracy studies report area under the receiver operator curve (AUC-ROC). Without accuracy estimates at specific cut-offs, such a result is difficult to apply. Some STARD group members felt that AUC-ROC is often overemphasized and argued that AUC-ROC can be difficult to appraise. Others mentioned that encouraging cut-offs could be misleading if these are arbitrary.

Should STARD recommend reporting at least one cut-off when reporting AUC-ROC?

- Yes
- No
- No opinion

Open comment box:

Terminology : “diagnostic accuracy” or “diagnostic performance”?

Considerations:

STARD refers at present to diagnostic accuracy, but “accuracy” is found to be a controversial term by some. Accuracy has other meanings, and an imperfect reference standard may represent only the best approximation to the "truth" of who has the target condition. “Diagnostic performance” is preferred by some, but this term is more general. Both terms are used in the literature.

Should we use “diagnostic accuracy” or “diagnostic performance” to refer to the results of comparisons of tests with a clinical reference standard?

- Keep “diagnostic accuracy”
- Change to “diagnostic performance”
- No opinion

Open comment box:

Open comments page.

Please feel free to comment on the STARD update process and to bring up any issues that were not discussed so far.
