# Supplementary material for: Updating standards for reporting diagnostic accuracy: the development of STARD 2015
Source: Res Integr Peer Rev. 2016 Jun 7;1:7. doi: 10.1186/s41073-016-0014-7 (PMC5803584; doi:10.1186/s41073-016-0014-7)
Supplement: Supplementary file 5 — Questionnaire for piloting among experts in the field of imaging. (PDF 83 kb) [file 41073_2016_14_MOESM5_ESM.pdf]

Welcome to the STARD update piloting survey.

STARD is a reporting guideline focusing on diagnostic accuracy studies.

It was first published in 2003 and endorsed by more than 200 journals.

The STARD Group is currently updating the checklist.

In this survey we would like to collect your feedback on this draft of the updated STARD checklist and the explanatory document that will constitute the flipside of the checklist.

Background information.

The results from this survey will be anonymized but you can leave your name if you like:

What is your main professional activity?

- ○ Clinical radiologist
- ○ Medical laboratory scientist
- ○ Physician
- ○ Biomedical researcher
- ○ Journal editor
- ○ Other

If other, please specify:

Were you ever co-author of a diagnostic accuracy study report?

- ○ Yes
- ○ No

STARD explanatory document

1. From this explanatory document, is the aim of STARD clear to you?

- Yes
- No
- No opinion

2. Do you feel that the information about STARD presented in this explanatory document is sufficient?

- Yes
- No
- No opinion

3. Do you feel that the terminology used in this explanatory document is easy to understand?

- Yes
- No
- No opinion

If "No" to any question, please elaborate:

STARD checklist: general comments

1. Are you satisfied with the layout of the checklist?

- Yes
- No
- No opinion

2. Are you satisfied with the outline of the checklist (headings and subheadings)?

- Yes
- No
- No opinion

3. Do you feel that the terminology used in the checklist is easy to understand?

- ○ Yes
- ○ No
- ○ No opinion

If "No" to any question, please elaborate:

STARD checklist: item-specific comments

1. Did you find any item(s) particularly difficult to understand?

- ○ Yes
- ○ No
- ○ No opinion

Please elaborate, if any items were difficult to you:

2. Do you feel that anything important is missing from the checklist?

- ◯ Yes
- ◯ No
- ◯ No opinion

Please elaborate, if you feel anything important is missing:

Open comments page.

Please feel free to provide any additional comment:
