# Supplementary material for: Updating standards for reporting diagnostic accuracy: the development of STARD 2015
Source: Res Integr Peer Rev. 2016 Jun 7;1:7. doi: 10.1186/s41073-016-0014-7 (PMC5803584; doi:10.1186/s41073-016-0014-7)
Supplement: Supplementary file 6 — Literature review: results. (DOCX 141 kb) [file 41073_2016_14_MOESM6_ESM.docx]

**Additional file 6. Literature review: results**

We extracted data relevant to the STARD update process following three categories:

1. general considerations about DTA studies and reporting;
2. suggestions for new items;
3. potential modification of existing items.
4. **General considerations**

| **Consideration** | **Rationale** | **References** |
| --- | --- | --- |
| Should STARD adopt the 4-domain structure of QUADAS-2? | QUADAS-2 is a 4-domain tool for assessing risk of bias and applicability concerns in systematic reviews of diagnostic accuracy studies. The domains are: patient selection, index test, reference standard, and flow & timing. | [1,2] |
| Should STARD provide a glossary defining key terms used in the list of essential items? | STROBE-ME, for example, provides a box with definition of key terms used in the text and checklist. | [3] |
| Should STARD provide a glossary defining different types of bias in diagnostic accuracy studies? | Definition of common types of bias in diagnostic accuracy studies could facilitate the understanding of key elements of reporting; this was done in the 2012 AHRQ report. | [4] |

1. **Suggestions for new items**

| **Section** | **Potential new item** | **References** |
| --- | --- | --- |
| INTRODUCTION | In the Background section, state frequency, morbidity and mortality of the target condition, and whether a treatment exists | [5] |
|  | State any prespecified study hypotheses | [6-8] |
|  | Describe the phase of evaluation | [9] |
| METHODS | Report the sample size calculations | [5-7,10-14] |
|  | Explicitly report the study design (single-gate vs. multiple-gate study) | [15] |
|  | In case of a composite reference standard, report how it was selected and the combination rule | [16] |
|  | Address potential sources of bias and statistical methods to correct for bias, if done | [17-20] |
| OTHER | Report where the study protocol can be accessed | [21,22] |
|  | Report the registration number | [21-26] |
|  | Report sources of funding | [8,22,27-31] |
|  | Report whether patient level data are available to share | [32] |

1. **Potential modification of existing 2003 STARD items**

| **STARD item #** | **Potential modification** | **References** |
| --- | --- | --- |
| 1 | Recommend keywords to use in the title and/or abstract to improve indexing and retrieval of diagnostic accuracy studies. GRIPS, for example, recommends to report the following keywords in the abstract: genetic, genomic, risk, prediction | [33] |
| 2 | Add background information about the clinical pathway, including the purpose and role of the index test, and the clinical context | [4,14,34] |
| 2 | Define all clinical end points and outcomes examined (because primary and secondary outcomes are rarely reported in diagnostic accuracy studies) | [7] |
| 2 | State clearly the diagnostic accuracy research question which the study aims to address | [32] |
| 4 | Report whether the study was a single-gate (cohort) or multiple-gate (case-control) study | [1,15,35] |
| 6 | Specify the relative order in which data collection, analysis planning, index test(s) and reference standard were performed (terms “prospective” and “retrospective” are often ambiguous) | [32] |
| 8 & 10 | Report details about collection, processing and storage of biological sample | [3,7] |
| 9 | Whether positivity thresholds (ie, cutoff values) were selected a priori | [1,35-37] |
| 9 | Rationale for the selected positivity thresholds | [38] |
| 13 & 24 | The term “reproducibility” many be too vague and require additional explanation | [35] |
| 15 | Be more specific about characteristics that should be reported, for example: age, sex, level of education, and spectrum of disease for participants, and characteristics of recruitment centers | [32] |
| 16 | Describe the flow of patients through the study, including the number of patients included in each stage of the analysis (a diagram may be helpful) and reasons for dropout. Specifically, both overall and for each subgroup extensively examined report the numbers of patients and the number of events.  There is large variability in the reporting of this element; a more extensive and specific description, like in STARDdem, may be helpful. | [32] |
| 17 | “Treatment” could be replaced by a more broader term, such as “intervention” | [1] |
| 17 | Describe the order of testing (e.g. index test first, then reference standard); timing of assessment of disease status and outcomes | [4,32] |
| 19 | Disease prevalence could be explicitly requested | [35,39,40] |
| 20 | This item could also address elements such as patient preferences, anxiety, pain, discomfort from testing | [14] |
| 23 | Assessment of variability of diagnostic accuracy could be extended to multivariable adjustment and modeling | [10,13,41] |
| 23 | State which subgroup analyses were pre-planned | [10,41] |
| 25 | Key findings and results | [27-30] |
| 25 | Potential sources of bias and imprecision | [8,27,35] |
| 25 | Applicability and generalizability of findings | [22,27,29] |
| 25 | Clinical and scientific context | [29,30,42] |
| 25 | Study limitations | [22,28-30,43] |
| 25 | Implications for clinical practice and patient management | [44] |
| 25 | Implications for future research | [7] |
| 25 | Interpretation and conclusion | [22,27,28] |

**References**

1. Whiting PF, Rutjes AW, Westwood ME, Mallett S, Deeks JJ, et al. (2011) QUADAS-2: a revised tool for the quality assessment of diagnostic accuracy studies. Ann Intern Med 155: 529-536.

2. Whiting PF, Rutjes AW, Westwood ME, Mallett S (2013) A systematic review classifies sources of bias and variation in diagnostic test accuracy studies. J Clin Epidemiol 66: 1093-1104.

3. Gallo V, Egger M, McCormack V, Farmer PB, Ioannidis JP, et al. (2011) STrengthening the Reporting of OBservational studies in Epidemiology--Molecular Epidemiology (STROBE-ME): an extension of the STROBE Statement. PLoS Med 8: e1001117.

4. Chang S, Matchar D (2012) Methods guide for medical test reviews. AHRQ publication No. 12-EC017. Agency for Healthcare Research and Quality, Rockville, MD.

5. Launay E, Morfouace M, Deneux-Tharaux C, Gras le-Guen C, Ravaud P, et al. (2014) Quality of reporting of studies evaluating time to diagnosis: a systematic review in paediatrics. Arch Dis Child 99: 244-250.

6. Vach W, Gerke O, Hoilund-Carlsen PF (2012) Three principles to define the success of a diagnostic study could be identified. J Clin Epidemiol 65: 293-300.

7. Altman DG, McShane LM, Sauerbrei W, Taube SE (2012) Reporting Recommendations for Tumor Marker Prognostic Studies (REMARK): explanation and elaboration. PLoS Med 9: e1001216.

8. Little J, Higgins JP, Ioannidis JP, Moher D, Gagnon F, et al. (2009) STrengthening the REporting of Genetic Association Studies (STREGA): an extension of the STROBE statement. PLoS Med 6: e22.

9. Lijmer JG, Leeflang M, Bossuyt PM (2009) Proposals for a phased evaluation of medical tests. Med Decis Making 29: E13-21.

10. Bachmann LM, Puhan MA, ter Riet G, Bossuyt PM (2006) Sample sizes of studies on diagnostic accuracy: literature survey. BMJ 332: 1127-1129.

11. Bochmann F, Johnson Z, Azuara-Blanco A (2007) Sample size in studies on diagnostic accuracy in ophthalmology: a literature survey. Br J Ophthalmol 91: 898-900.

12. Flahault A, Cadilhac M, Thomas G (2005) Sample size calculation should be performed for design accuracy in diagnostic test studies. J Clin Epidemiol 58: 859-862.

13. Knottnerus JA, Muris JW (2003) Assessment of the accuracy of diagnostic tests: the cross-sectional study. J Clin Epidemiol 56: 1118-1128.

14. Gatsonis C (2012) Standards in the design, conduct and evaluation of diagnostic testing for use in patient centered outcomes research. pp. 59.

15. Rutjes AW, Reitsma JB, Vandenbroucke JP, Glas AS, Bossuyt PM (2005) Case-control and two-gate designs in diagnostic accuracy studies. Clin Chem 51: 1335-1341.

16. Naaktgeboren CA, Bertens LC, van Smeden M, de Groot JA, Moons KG, et al. (2013) Value of composite reference standards in diagnostic research. BMJ 347: f5605.

17. Bachmann LM, ter Riet G, Weber WE, Kessels AG (2009) Multivariable adjustments counteract spectrum and test review bias in accuracy studies. J Clin Epidemiol 62: 357-361 e352.

18. de Groot JA, Dendukuri N, Janssen KJ, Reitsma JB, Bossuyt PM, et al. (2011) Adjusting for differential-verification bias in diagnostic-accuracy studies: a Bayesian approach. Epidemiology 22: 234-241.

19. de Groot JA, Janssen KJ, Zwinderman AH, Bossuyt PM, Reitsma JB, et al. (2011) Correcting for partial verification bias: a comparison of methods. Ann Epidemiol 21: 139-148.

20. Cronin AM, Vickers AJ (2008) Statistical methods to correct for verification bias in diagnostic studies are inadequate when there are few false negatives: a simulation study. BMC Med Res Methodol 8: 75.

21. Ioannidis JP, Greenland S, Hlatky MA, Khoury MJ, Macleod MR, et al. (2014) Increasing value and reducing waste in research design, conduct, and analysis. Lancet 383: 166-175.

22. Schulz KF, Altman DG, Moher D, Group C (2010) CONSORT 2010 statement: updated guidelines for reporting parallel group randomised trials. PLoS Med 7: e1000251.

23. Hooft L, Bossuyt PM (2011) Prospective registration of marker evaluation studies: time to act. Clin Chem 57: 1684-1686.

24. Korevaar DA, Bossuyt PM, Hooft L (2014) Infrequent and incomplete registration of test accuracy studies: analysis of recent study reports. BMJ Open 4: e004596.

25. Rifai N, Bossuyt PM, Ioannidis JP, Bray KR, McShane LM, et al. (2014) Registering diagnostic and prognostic trials of tests: is it the right thing to do? Clin Chem 60: 1146-1152.

26. Walker KF, Stevenson G, Thornton JG (2014) Discrepancies between registration and publication of randomised controlled trials: an observational study. JRSM Open 5: 2042533313517688.

27. von Elm E, Altman DG, Egger M, Pocock SJ, Gotzsche PC, et al. (2007) The Strengthening the Reporting of Observational Studies in Epidemiology (STROBE) statement: guidelines for reporting observational studies. PLoS Med 4: e296.

28. Moher D, Liberati A, Tetzlaff J, Altman DG, Group P (2009) Preferred reporting items for systematic reviews and meta-analyses: the PRISMA statement. PLoS Med 6: e1000097.

29. Ogrinc G, Mooney SE, Estrada C, Foster T, Goldmann D, et al. (2008) The SQUIRE (Standards for QUality Improvement Reporting Excellence) guidelines for quality improvement reporting: explanation and elaboration. Qual Saf Health Care 17 Suppl 1: i13-32.

30. Husereau D, Drummond M, Petrou S, Carswell C, Moher D, et al. (2013) Consolidated Health Economic Evaluation Reporting Standards (CHEERS) statement. BMJ 346: f1049.

31. Polyzos NP, Valachis A, Mauri D, Ioannidis JP (2011) Industry involvement and baseline assumptions of cost-effectiveness analyses: diagnostic accuracy of the Papanicolaou test. CMAJ 183: E337-343.

32. Noel-Storr AH, McCleery JM, Richard E, Ritchie CW, Flicker L, et al. (2014) Reporting standards for studies of diagnostic test accuracy in dementia: The STARDdem Initiative. Neurology 83: 364-373.

33. Janssens AC, Ioannidis JP, Bedrosian S, Boffetta P, Dolan SM, et al. (2011) Strengthening the reporting of genetic risk prediction studies (GRIPS): explanation and elaboration. Eur J Clin Invest 41: 1010-1035.

34. Horvath AR, Lord SJ, StJohn A, Sandberg S, Cobbaert CM, et al. (2014) From biomarkers to medical tests: the changing landscape of test evaluation. Clin Chim Acta 427: 49-57.

35. Whiting PF, Rutjes AW, Westwood ME, Mallett S, Group Q-S (2013) A systematic review classifies sources of bias and variation in diagnostic test accuracy studies. J Clin Epidemiol 66: 1093-1104.

36. Leeflang MM, Moons KG, Reitsma JB, Zwinderman AH (2008) Bias in sensitivity and specificity caused by data-driven selection of optimal cutoff values: mechanisms, magnitude, and solutions. Clin Chem 54: 729-737.

37. Ewald B (2006) Post hoc choice of cut points introduced bias to diagnostic research. J Clin Epidemiol 59: 798-801.

38. Tzoulaki I, Liberopoulos G, Ioannidis JP (2011) Use of reclassification for assessment of improved prediction: an empirical evaluation. Int J Epidemiol 40: 1094-1105.

39. Leeflang MM, Bossuyt PM, Irwig L (2009) Diagnostic test accuracy may vary with prevalence: implications for evidence-based diagnosis. J Clin Epidemiol 62: 5-12.

40. Leeflang MM, Rutjes AW, Reitsma JB, Hooft L, Bossuyt PM (2013) Variation of a test's sensitivity and specificity with disease prevalence. CMAJ 185: E537-544.

41. Janes H, Pepe MS (2008) Adjusting for covariates in studies of diagnostic, screening, or prognostic markers: an old concept in a new setting. Am J Epidemiol 168: 89-97.

42. Glasziou P, Altman DG, Bossuyt P, Boutron I, Clarke M, et al. (2014) Reducing waste from incomplete or unusable reports of biomedical research. Lancet 383: 267-276.

43. Ioannidis JP (2007) Limitations are not properly acknowledged in the scientific literature. J Clin Epidemiol 60: 324-329.

44. Staub LP, Lord SJ, Simes RJ, Dyer S, Houssami N, et al. (2012) Using patient management as a surrogate for patient health outcomes in diagnostic test evaluation. BMC Med Res Methodol 12: 12.
