# Supplementary material for: Updating standards for reporting diagnostic accuracy: the development of STARD 2015
Source: Res Integr Peer Rev. 2016 Jun 7;1:7. doi: 10.1186/s41073-016-0014-7 (PMC5803584; doi:10.1186/s41073-016-0014-7)
Supplement: Supplementary file 7 — Summary of responses to the online surveys. (DOCX 31 kb) [file 41073_2016_14_MOESM7_ESM.docx]

**Additional file 7. Summary of responses to the online survey**

1. **Responses to survey 1, Part 1: Existing items**

73/85 (86%) STARD group members responded. Items in green represent a majority response (≥75%).

| **Item 1 on STARD 2003 checklist**  **Question: Should we:** | |
| --- | --- |
| Keep this item as it is | 29.2% |
| Modify this item: provide more guidance on which terms to use in the title and abstract | 36.1% |
| Modify this item otherwise (please explain) | 4.2% |
| Remove this item (our suggestion) | 27.8% |
| No opinion | 2.8% |

| **Item 2 on STARD 2003 checklist**  **Question: Should we:** | |
| --- | --- |
| Keep this item as it is | 13.9% |
| Modify this item: invite authors to report the purpose, clinical context, and clinical role of the test (our suggestion) | 79.2% |
| Modify this item otherwise (please explain) | 6.9% |
| Remove this item | 0.0% |
| No opinion | 0.0% |

| **Item 3 on STARD 2003 checklist**  **Question: Should we:** | |
| --- | --- |
| Keep this item as it is | 16.7% |
| Modify this item: move “setting and locations” from item 3 to item 4 (“participant recruitment”) (our suggestion) | 68.1% |
| Modify this item otherwise (please explain) | 12.5% |
| Remove this item | 1.4% |
| No opinion | 1.4% |

| **Item 4 on STARD 2003 checklist**  **Question: Should we:** |  |
| --- | --- |
| Keep this item as it is | 9.9% |
| Modify this item: reword and simplify (our suggestion) | 78.9% |
| Modify this item otherwise (please explain) | 9.9% |
| Remove this item | 1.4% |
| No opinion | 0.0% |

| **Item 5 on STARD 2003 checklist**  **Question: Should we:** |  |
| --- | --- |
| Keep this item as it is (our suggestion) | 85.9% |
| Modify this item (please explain) | 11.3% |
| Remove this item | 2.8% |
| No opinion | 0.0% |

| **Item 6 on STARD 2003 checklist**  **Question: Should we:** |  |
| --- | --- |
| Keep this item as it is | 11.3% |
| Modify this item: reword and simplify (our suggestion) | 76.1% |
| Modify this item otherwise (please explain) | 8.5% |
| Remove this item | 4.2% |
| No opinion | 0.0% |

| **Item 7 on STARD 2003 checklist**  **Question: Should we:** |  |
| --- | --- |
| Keep this item as it is | 22.5% |
| Modify this item: remove "and its rationale" (our suggestion) | 54.9% |
| Modify this item otherwise (please explain) | 21.1% |
| Remove this item | 0.0% |
| No opinion | 1.4% |

| **Item 8 on STARD 2003 checklist**  **Question: Should we:** |  |
| --- | --- |
| Keep this item as it is | 8.5% |
| Modify this item: refer to list of preferred descriptions for specific test types (to be developed) (our suggestion) | 84.5% |
| Modify this item otherwise (please explain) | 4.2% |
| Remove this item | 0.0% |
| No opinion | 2.8% |

| **Item 9 on STARD 2003 checklist**  **Question: Should we:** |  |
| --- | --- |
| Keep this item as it is | 1.4% |
| Modify this item: remove “units” and invite authors to report whether cut-offs and/or categories were pre-specified (our suggestion) | 77.5% |
| Modify this item otherwise (please explain) | 18.3% |
| Remove this item | 2.8% |
| No opinion | 0.0% |

| **Item 10 on STARD 2003 checklist**  **Question: Should we:** |  |
| --- | --- |
| Keep this item as it is (our suggestion) | 81.7% |
| Modify this item (please explain) | 16.9% |
| Remove this item | 1.4% |
| No opinion | 0.0% |

| **Item 11 on STARD 2003 checklist**  **Question: Should we:** |  |
| --- | --- |
| Keep this item as it is | 4.2% |
| Modify this item: reword and simplify (our suggestion) | 84.5% |
| Modify this item otherwise (please explain) | 11.3% |
| Remove this item | 0.0% |
| No opinion | 0.0% |

| **Item 12 on STARD 2003 checklist**  **Question: Should we:** |  |
| --- | --- |
| Keep this item as it is | 9.9% |
| Modify this item: reword and simplify (our suggestion) | 78.9% |
| Modify this item otherwise (please explain) | 11.3% |
| Remove this item | 0.0% |
| No opinion | 0.0% |

| **Item 13 on STARD 2003 checklist**  **Question: Should we:** |  |
| --- | --- |
| Keep this item as it is | 1.4% |
| Modify this item (please explain) | 14.1% |
| Remove this item, and integrate in item 8 (“technical specifications”) (our suggestion) | 76.1% |
| Remove this item | 7.0% |
| No opinion | 1.4% |

| **Item 14 on STARD 2003 checklist**  **Question: Should we:** |  |
| --- | --- |
| Keep this item as it is | 9.9% |
| Modify this item (please explain) | 4.2% |
| Remove this item, and integrate in item 4 (“participant recruitment”) (our suggestion) | 81.7% |
| Remove this item | 2.8% |
| No opinion | 1.4% |

| **Item 15 on STARD 2003 checklist**  **Question: Should we:** |  |
| --- | --- |
| Keep this item as it is | 25.4% |
| Modify this item: simplify and remove proposed characteristics (our suggestion) | 60.6% |
| Modify this item otherwise (please explain) | 12.7% |
| Remove this item | 1.4% |
| No opinion | 0.0% |

| **Item 16 on STARD 2003 checklist**  **Question: Should we:** |  |
| --- | --- |
| Keep this item as it is | 9.9% |
| Modify this item: reword and always require a flow diagram (our suggestion) | 84.5% |
| Modify this item otherwise (please explain) | 5.6% |
| Remove this item | 0.0% |
| No opinion | 0.0% |

| **Item 17 on STARD 2003 checklist**  **Question: Should we:** |  |
| --- | --- |
| Keep this item as it is (our suggestion) | 90.1% |
| Modify this item (please explain) | 8.5% |
| Remove this item | 0.0% |
| No opinion | 1.4% |

| **Item 18 on STARD 2003 checklist**  **Question: Should we:** |  |
| --- | --- |
| Keep this item as it is (our suggestion) | 90.1% |
| Modify this item (please explain) | 9.9% |
| Remove this item | 0.0% |
| No opinion | 0.0% |

| **Item 19 on STARD 2003 checklist**  **Question: Should we:** |  |
| --- | --- |
| Keep this item as it is | 8.5% |
| Modify this item: simplify and remove the terms “including indeterminate and missing results” (our suggestion) | 76.1% |
| Modify this item otherwise (please explain) | 14.1% |
| Remove this item | 1.4% |
| No opinion | 0.0% |

| **Item 20 on STARD 2003 checklist**  **Question: Should we:** |  |
| --- | --- |
| Keep this item as it is | 45.1% |
| Modify this item (please explain) | 9.9% |
| Remove this item (our suggestion) | 40.8% |
| No opinion | 4.2% |

| **Item 21 on STARD 2003 checklist**  **Question: Should we:** |  |
| --- | --- |
| Keep this item as it is (our suggestion) | 90.1% |
| Modify this item (please explain) | 8.5% |
| Remove this item | 1.4% |
| No opinion | 0.0% |

| **Item 22 on STARD 2003 checklist**  **Question: Should we:** |  |
| --- | --- |
| Keep this item as it is | 8.5% |
| Modify this item: move to the “methods” items (our suggestion) | 78.9% |
| Modify this item otherwise (please explain) | 11.3% |
| Remove this item | 0.0% |
| No opinion | 1.4% |

| **Item 23 on STARD 2003 checklist**  **Question: Should we:** |  |
| --- | --- |
| Keep this item as it is | 8.5% |
| Modify this item: invite authors to report whether subgroup analyses were pre-planned (our suggestion) | 69.0% |
| Modify this item otherwise (please explain) | 19.7% |
| Remove this item | 1.4% |
| No opinion | 1.4% |

| **Item 24 on STARD 2003 checklist**  **Question: Should we:** |  |
| --- | --- |
| Keep this item as it is | 8.5% |
| Modify this item (please explain) | 5.6% |
| Remove this item, and integrate in item 8 (“technical specifications of index test and reference standard”) (our suggestion) | 80.3% |
| Remove this item | 5.6% |
| No opinion | 0.0% |

| **Item 25 on STARD 2003 checklist**  **Question: Should we:** |  |
| --- | --- |
| Keep this item as it is | 26.8% |
| Modify this item (please explain) | 43.7% |
| Remove this item | 26.8% |
| No opinion | 2.8% |

1. **Responses to survey 1, Part 2: Potential new items**

73/85 (86%) STARD group members responded. Items in green represent a majority response (≥75%).

|  | | **Yes** | **No** | **No opinion** |
| --- | --- | --- | --- | --- |
| Should STARD recommend reporting the method and rationale for the study sample size calculation? | | 78,1% | 19,2% | 2,7% |
| Should STARD recommend reporting at least one cut-off when reporting AUC-ROC? | | 68,5% | 23,3% | 8,2% |
| Should STARD recommend reporting... | |  |  |  |
|  | ...the trial registration number? | 74,0% | 11,0% | 15,1% |
|  | ...a link to online resources with more information on the study? | 74,0% | 9,6% | 15,1% |
|  | ...about the availability of the study protocol? | 83,6% | 9,6% | 6,8% |
|  | ...about the availability of patient level data, or the data sharing policy? | 68,5% | 13,7% | 17,8% |
|  | ...conflicts of interest? | 87,7% | 9,6% | 2,7% |
|  | ...sources of funding? | 89,0% | 6,8% | 4,1% |
| Should the applicability of STARD be rephrased, from “diagnostic accuracy studies” to “studies reporting diagnostic accuracy”? | | 65,8% | 17,8% | 16,4% |
| Should the applicability of STARD be extended to prognostic accuracy studies? | | 61,6% | 21,9% | 16,4% |
| Should the applicability of STARD be rephrased, from “diagnostic accuracy” to “(clinical) test accuracy”? | | 47,9% | 32,9% | 19,2% |
| Should the applicability of STARD be rephrased, e.g. in terms of “all evaluations of the accuracy of one or more tests, or combinations of test results and/or other variables” | | 79,5% | 13,7% | 6,8% |
| Should STARD recommend preferred terms for indicating... | |  |  |  |
| ...the type of study (e.g. “a diagnostic accuracy study” or “a test accuracy study”)? | | 68,5% | 20,5% | 11,0% |
| ...the study design (e.g. cohort/case-control or single-gate/multiple-gate studies)? | | 61,6% | 27,4% | 11,0% |
| ...the “index test” and the “clinical reference standard”? | | 80,8% | 13,7% | 5,5% |

1. **Responses to survey 2: Elements that did not receive a majority vote in survey 1**

79/85 (93%) STARD group members responded. Items in green represent a majority response (≥75%).

| **Item 3 on STARD 2003 checklist**  **Question: Should we:** | |
| --- | --- |
| Keep item 3 as it is | 29,1% |
| Reword item 3 into “Study population - Describe the inclusion and exclusion criteria” (“setting and locations” to be included in item 4, under “Describe participant recruitment”) | 49,4% |
| No opinion | 21,5% |

| **Item 7 on STARD 2003 checklist**  **Question: Should we:** | |
| --- | --- |
| Keep item 7 as it is | 12,7% |
| Modify item 7 into “Describe the reference standard” | 20,3% |
| Modify item 7: reword to make clear that “rationale” refers to the choice of a reference standard among alternatives | 65,8% |
| No opinion | 1,3% |

| **Item 15 on STARD 2003 checklist**  **Question: Should we:** | |
| --- | --- |
| Keep item 15 as it is | 38% |
| Modify item 15 into “Report demographic and clinical characteristics of the study participants” and provide more guidance in the explanatory document | 59,5% |
| No opinion | 2,5% |

| **Item 20 on STARD 2003 checklist**  **Question: Should we:** | |
| --- | --- |
| Keep item 20 as it is | 82,3% |
| Remove item 20 | 13,9% |
| No opinion | 3,8% |

| **Item 23 on STARD 2003 checklist**  **Question: Should we:** | |
| --- | --- |
| Modify item 23: invite authors to report in the methods whether subgroup analyses were preplanned | 16,5% |
| Modify item 23: invite authors to report in the methods whether subgroup analyses were preplanned and provide guidance on how to report subgroup differences in the explanatory document | 81% |
| No opinion | 2,5% |

| **Item 25 on STARD 2003 checklist**  **Question: Should we:** | |
| --- | --- |
| Keep item 25 as it is | 46,8% |
| Modify item 25: invite authors to adopt a structured discussion | 34,2% |
| Remove this item | 12,7% |
| No opinion | 6,3% |

| **Potential new item**  **Question: Should STARD recommend reporting at least one cut-off when reporting AUC-ROC?** | |
| --- | --- |
| Yes | 55,7% |
| No | 31,6% |
| No opinion | 12,7% |

| **Wording**  **Question: Should we use “diagnostic accuracy” or “diagnostic performance” to refer to the results of comparisons of tests with a clinical reference standard?** | |
| --- | --- |
| Keep “diagnostic accuracy” | 64,6% |
| Change to “diagnostic performance” | 17,7% |
| No opinion | 17,7% |
