# Supplementary material for: Updating standards for reporting diagnostic accuracy: the development of STARD 2015
Source: Res Integr Peer Rev. 2016 Jun 7;1:7. doi: 10.1186/s41073-016-0014-7 (PMC5803584; doi:10.1186/s41073-016-0014-7)
Supplement: Supplementary file 8 — Feedback from editorial board members and reviewers from Radiology. (DOCX 28 kb) [file 41073_2016_14_MOESM8_ESM.docx]

**Additional file 8. Feedback from editorial board members and reviewers from Radiology (N=20)**

|  | | **Yes** | **No** | **No opinion** | **Comments** |
| --- | --- | --- | --- | --- | --- |
| **Flipside** | | | | | |
|  | From this explanatory document, is the aim of STARD clear to you? | 18 | 1 | 1 | - |
|  | Do you feel that the information about STARD presented in this explanatory document is sufficient? | 17 | 2 | 1 | - |
|  | Do you feel that the terminology used in this explanatory document is easy to understand? | 16 | 2 | 2 | 2 |
| **Checklist** | | | | | |
|  | Are you satisfied with the layout of the checklist? | 16 | 3 | 1 | - |
|  | Are you satisfied with the outline of the checklist (headings and subheadings)? | 18 | 1 | 1 | - |
|  | Do you feel that the terminology used in the checklist is easy to understand? | 17 | 2 | 1 | 4 |
|  | Did you find any item(s) particularly difficult to understand?* | 4 | 15 | 0 | 4 |
|  | Do you feel that anything important is missing from the checklist?** | 2 | 15 | 1 | 2 |

*1 missing value; **2 missing values.
