# Supplementary material for: Updating standards for reporting diagnostic accuracy: the development of STARD 2015
Source: Res Integr Peer Rev. 2016 Jun 7;1:7. doi: 10.1186/s41073-016-0014-7 (PMC5803584; doi:10.1186/s41073-016-0014-7)
Supplement: Supplementary file 9 — Rationale for STARD 2015 items. (DOCX 45 kb) [file 41073_2016_14_MOESM9_ESM.docx]

**Additional file 9. Rationale for STARD 2015 items**

**1. Rationale for changes from STARD 2003 to STARD 2015 items**

|  | **Item #** | **Item description** |
| --- | --- | --- |
| STARD 2003 | 1 | Identify the article as a study of diagnostic accuracy (recommend MeSH heading 'sensitivity and specificity'). |
| STARD 2015 | 1 | Identification as a study of diagnostic accuracy using at least one measure of accuracy (such as sensitivity, specificity, predictive values, or AUC) |
| *Survey results* |  | **Survey 1**: 28% agreed with our suggestion to remove the item, while 36% preferred to modify the item by providing more guidance on which terms to use in the title and abstract, and 29% suggested to keep the item as it is **(*no majority response*)**. |
| *Rationale for change* |  | A wide variety of terms is used to announce studies of diagnostic accuracy; “diagnostic accuracy” itself is not frequently used. Authors do not decide on MeSH headings, and the previously recommended MeSH heading ‘sensitivity and specificity’ has shown to be very nonsensitive when searching for diagnostic accuracy studies. Search filters for diagnostic accuracy studies contain measures of accuracy; using such a term in title or abstract would facilitate identification. |
| STARD 2003 | 2 | State the research questions or study aims, such as estimating diagnostic accuracy or comparing accuracy between tests or across participant groups. |
| STARD 2015 | 4 | Study objectives and hypotheses |
| *Survey results* |  | **Survey 1**: 79% agreed with our suggestion to invite authors to report the purpose, clinical context, and clinical role of the test **(*majority response*)**. |
| *Rationale for change* |  | Many diagnostic accuracy studies report vague and very general study questions/aims. Prespecifying study hypotheses should be stimulated. This item is also part of CONSORT. In the new item 3 (see “Rationale for new items in STARD 2015” table below), we now also recommend authors to report on the scientific and clinical background, including the intended use and clinical role of the index test, before providing the study objectives and hypotheses. |
| STARD 2003 | 3 | Describe the study population: The inclusion and exclusion criteria, setting and locations where the data were collected. |
| STARD 2015 | 6 | Eligibility criteria |
|  | 8 | Where and when potentially eligible participants were identified (setting, location and dates) |
| *Survey results* |  | **Survey 1**: 68% agreed with our suggestion to remove “setting and locations” from this item and integrate it with the item about “participant recruitment” (item 4 on STARD 2003 list) **(*no majority response*)**.  **Survey 2**: 49% agreed with our suggestion to remove “setting and locations” from this item and integrate it with the item about “participant recruitment” (item 4 on STARD 2003 list) **(*no majority response*)**. |
| *Rationale for change* |  | Setting and locations are features of participant recruitment and should be separate from the inclusion and exclusion criteria. It was decided to make a separate item for setting and locations, and to not include it with either “study population” (item 3 on STARD 2003 list) or “participant recruitment” (item 4 on STARD 2003 list). This is in line with CONSORT, which also separates these two items. |
| STARD 2003 | 4 | Describe participant recruitment: Was recruitment based on presenting symptoms, results from previous tests, or the fact that the participants had received the (evaluated) index tests or the (golden) reference standard? |
| STARD 2015 | 7 | On what basis potentially eligible participants were identified (such as symptoms, results from previous tests, inclusion in registry) |
| *Survey results* |  | **Survey 1**: 79% agreed with our suggestion to reword and simplify the item **(*majority response*)**. |
| *Rationale for change* |  | “Participant recruitment” is a vague term, and sometimes led to misinterpretation of the item, so this term was avoided. |
| STARD 2003 | 5 | Describe participant sampling: Was the study population a consecutive series of participants defined by the selection criteria in items 3 and 4? If not, specify how participants were further selected. |
| STARD 2015 | 9 | Whether participants formed a consecutive, random, or convenience series |
| *Survey results* |  | **Survey 1**: 86% agreed with our suggestion to keep this item as it is **(*majority response*)**. |
| *Rationale for change* |  | During the Steering Committee Meeting there were concerns that the term “sampling” is confusing because it may refer to test samples, leading to some minor rewording in which this term was avoided. |
| STARD 2003 | 6 | Describe data collection: Was data collection planned before the index test and reference standard were performed (prospective study) or after (retrospective study)? |
| STARD 2015 | 5 | Whether data collection was planned before the index test and reference standard were performed (prospective study) or after (retrospective study) |
| *Survey results* |  | **Survey 1**: 76% agreed with our suggestion to reword and simplify the item **(*majority response*)**. |
| *Rationale for change* |  | The general consensus in the STARD group is that it is relevant to know in which order question formulation and data collection took place. There is widespread variability in the interpretation of the labels “prospective” and “retrospective”, so we felt that these terms should be avoided and proposed to the STARD group to reword and simply the item. Rewording of this item turned out to further complicate this item during the piloting sessions. Therefore, we decided to keep the item as it is. |
| STARD 2003 | 7 | Describe the reference standard and its rationale. |
| STARD 2015 | 10b | Reference standard, in sufficient detail to allow replication |
|  | 11 | Rationale for choosing the reference standard (if alternatives exist) |
| *Survey results* |  | **Survey 1**: 55% agreed with our suggestion to remove “and its rationale”, while 23% preferred to keep the item as it is, and 21% suggested to modify it differently **(*no majority response*)**.  **Survey 2**: 66% agreed with our suggestion to make clear in this item that the “rationale” refers to the choice of a reference standard among alternatives **(*no majority response*)**. |
| *Rationale for change* |  | The rationale of a reference standard is often not reported in diagnostic accuracy studies, because it is only (but very) relevant if alternatives exist. |
| STARD 2003 | 8 | Describe technical specifications of material and methods involved including how and when measurements were taken, and/or cite references for index tests and reference standard. |
| STARD 2015 | 10a | Index test, in sufficient detail to allow replication |
|  | 10b | Reference standard, in sufficient detail to allow replication |
| *Survey results* |  | **Survey 1**: 85% agreed with our suggestion to refer to a list of preferred descriptions for specific test types **(*majority response*)**. |
| *Rationale for change* |  | Technical specifications vary widely across research fields (eg radiology and clinical chemistry): STARD extensions will be developed for each of these, describing the specific technical specifications that should be reported for tests in that specific field of research. |
| STARD 2003 | 9 | Describe definition of and rationale for the units, cut-offs and/or categories of the results of the index tests and the reference standard. |
| STARD 2015 | 12a | Definition of and rationale for test positivity cut-offs or result categories of the index test, distinguishing pre-specified from exploratory |
|  | 12b | Definition of and rationale for test positivity cut-offs or result categories of the reference standard, distinguishing pre-specified from exploratory |
| *Survey results* |  | **Survey 1**: 78% agreed with our suggestion to remove “units” and to invite authors to report whether cut-offs and/or categories were pre-specified **(*majority response*)**. |
| *Rationale for change* |  | Accuracy does not depend on the unit of measurement, but may change with the cutoffs and categories chosen to classify test results. Data-driven selection of a cutoff may lead to overoptimistic accuracy estimates. It is therefore useful to know whether cut-offs were pre-specified or not. A signaling question in QUADAS-2 is “If a threshold was used, was it pre-specified?”. This questions can only be answered if this information is reported. |
| STARD 2003 | 10 | Describe the number, training and expertise of the persons executing and reading the index tests and the reference standard. |
| STARD 2015 | 10a | Index test, in sufficient detail to allow replication |
|  | 10b | Reference standard, in sufficient detail to allow replication |
| *Survey results* |  | ­**Survey 1**: 82% agreed with our suggestion to keep this item as it is **(*majority response*)**. |
| *Rationale for change* |  | ­During the Steering Committee meeting it was argued that this item is not always applicable, especially in the field of laboratory medicine, and that this item is a feature of the specifications of the index test and reference standard and should be reported with those items. STARD extensions will be developed for different research fields, describing the specific technical specifications that should be reported for tests in that specific field of research. |
| STARD 2003 | 11 | Describe whether or not the readers of the index tests and reference standard were blind (masked) to the results of the other test and describe any other clinical information available to the readers. |
| STARD 2015 | 13a | Whether clinical information and reference standard results were available to the performers or readers of the index test |
|  | 13b | Whether clinical information and index test results were available to the assessors of the reference standard |
| *Survey results* |  | **Survey 1**: 85% agreed with our suggestion to reword and simplify the item **(*majority response*)**. |
| *Rationale for change* |  | The original item contained both a negative statement (“blinding”) and a positive statement (“clinical information available”) and, therefore, it was decided to reword. There is widespread variability in the interpretation of the label “blind”. It is important to know which specific clinical information is available to the readers of the tests, because this may influence the interpretation of the test result. |
| STARD 2003 | 12 | Describe methods for calculating or comparing measures of diagnostic accuracy, and the statistical methods used to quantify uncertainty (e.g. 95% confidence intervals). |
| STARD 2015 | 14 | Methods for estimating or comparing measures of diagnostic accuracy |
| *Survey results* |  | **Survey 1**: 79% agreed with our suggestion to reword and simplify the item **(*majority response*)**. |
| *Rationale for change* |  | The nature of statistical methods to be reported seems unclear to many authors: methods for the accuracy statistics, or for the uncertainty, or both? |
| STARD 2003 | 13 | Describe methods for calculating test reproducibility, if done. |
| STARD 2015 | 10a | Index test, in sufficient detail to allow replication |
|  | 10b | Reference standard, in sufficient detail to allow replication |
| *Survey results* |  | **Survey 1**: 76% agreed with our suggestion to remove the item and integrate it with the item about “technical specifications” (item 8 on the STARD 2003 list) **(*majority response*)**. |
| *Rationale for change* |  | The word “reproducibility” is ambiguous. Estimating a test’s reproducibility is not an element of most diagnostic accuracy studies. Many studies refer to other publications or to the manufacturer for information on test reproducibility, but this information should be part of the item about test specifications. |
| STARD 2003 | 14 | Report when study was done, including beginning and ending dates of recruitment. |
| STARD 2015 | 8 | Where and when potentially eligible participants were identified (setting, location, and dates) |
| *Survey results* |  | **Survey 1**: 82% agreed with our suggestion to integrate this the in the methods section with the item about “participant recruitment” (item 4 on the STARD 2003 list) **(*majority response*)**. |
| *Rationale for change* |  | STARD evaluations have shown that this item is almost always reported in the methods section, rarely in the results section. The item refers to participant recruitment. |
| STARD 2003 | 15 | Report clinical and demographic characteristics of the study population (e.g. age, sex, spectrum of presenting symptoms, co morbidity, current treatments, recruitment centers). |
| STARD 2015 | 20 | Baseline demographic and clinical characteristics of participants |
| *Survey results* |  | **Survey 1**: 61% agreed with our suggestion to simplify the item and remove the proposed characteristics, while 25% preferred to keep the item as it is **(*no majority response*)**.  **Survey 2**: 60% agreed with our suggestion to modify the item as “Report demographic and clinical characteristics of the study participants” and provide more guidance in the explanatory document, while 38% preferred to keep the item as it is **(*no majority response*)**. |
| *Rationale for change* |  | Depending on the type of test and target condition, there is a very large variety in suitable clinical and demographic characteristics reported in diagnostic accuracy studies. Therefore, it was decided to remove the examples, but provide guidance in the explanatory document. We used the same language as used in CONSORT. |
| STARD 2003 | 16 | Report the number of participants satisfying the criteria for inclusion that did or did not undergo the index tests and/or the reference standard; describe why participants failed to receive either test (a flow diagram is strongly recommended). |
| STARD 2015 | 19 | Flow of participants, using a diagram |
| *Survey results* |  | ­**Survey 1**: 85% agreed with our suggestion to reword the item and always require a flow diagram **(*majority response*)**. |
| *Rationale for change* |  | The item was lengthy and complex and often misunderstood. The reporting of flow diagrams, rarely reported by diagnostic accuracy studies, should be stimulated because such a diagram provides the most complete information with regard to the flow of patients through a study. |
| STARD 2003 | 17 | Report time interval from the index tests to the reference standard, and any treatment administered between. |
| STARD 2015 | 22 | Time interval and any clinical interventions between index test and reference standard |
| *Survey results* |  | **Survey 1**: 90% agreed with our suggestion to keep the item as it is **(*majority response*)**. |
| *Rationale for change* |  | *not applicable* |
| STARD 2003 | 18 | Report distribution of severity of disease (define criteria) in those with the target condition; other diagnoses in participants without the target condition. |
| STARD 2015 | 21a | Distribution of severity of disease in those with the target condition |
|  | 21b | Distribution of alternative diagnoses in those without the target condition |
| *Survey results* |  | **Survey 1**: 90% agreed with our suggestion to keep this item is at is **(*majority response*)**. |
| *Rationale for change* |  | *not applicable* |
| STARD 2003 | 19 | Report a cross tabulation of the results of the index tests (including indeterminate and missing results) by the results of the reference standard; for continuous results, the distribution of the test results by the results of the reference standard. |
| STARD 2015 | 23 | Cross tabulation of the index test results (or their distribution) by the results of the reference standard |
| *Survey results* |  | **Survey 1**: 76% agreed with our suggestion to simplify the item and remove the terms “including indeterminate and missing results” **(*majority response*)**. |
| *Rationale for change* |  | Indeterminate and missing results are almost never reported in cross tabulations. Item 22 on the STARD 2003 list already addresses how indeterminate and missing results are handled. |
| STARD 2003 | 20 | Report any adverse events from performing the index tests or the reference standard. |
| STARD 2015 | 25 | Any adverse events from performing the index test or the reference standard |
| *Survey results* |  | **Survey 1**: 41% agreed with our suggestion to remove the item, while 45% preferred to keep the item as it is **(*no majority response*)**.  **Survey 2**: 82% preferred to keep the item as it is **(*majority response*)**. |
| *Rationale for change* |  | *not applicable* |
| STARD 2003 | 21 | Report estimates of diagnostic accuracy and measures of statistical uncertainty (e.g. 95% confidence intervals). |
| STARD 2015 | 24 | Estimates of diagnostic accuracy and their precision (such as 95% confidence intervals) |
| *Survey results* |  | ­**Survey 1**: 90% agreed with our suggestion to keep the item as it is **(*majority response*)**. |
| *Rationale for change* |  | *not applicable* |
| STARD 2003 | 22 | Report how indeterminate results, missing responses and outliers of the index tests were handled. |
| STARD 2015 | 15 | How indeterminate index test or reference standard results were handled |
|  | 16 | How missing data on the index test and reference standard were handled |
| *Survey results* |  | **Survey 1**: 79% agreed with our suggestion to move the item to the “methods” items **(*majority response*)**. |
| *Rationale for change* |  | Moved to methods section because authors should be encouraged to plan ahead how to handle indeterminate results, missing responses and outliers in their study protocol. |
| STARD 2003 | 23 | Report estimates of variability of diagnostic accuracy between subgroups of participants, readers or centers, if done. |
| STARD 2015 | 17 | Any analyses of variability in diagnostic accuracy, distinguishing pre-specified from exploratory |
| *Survey results* |  | **Survey 1**: 69% agreed with our suggestion to invite authors to report whether subgroup analyses were preplanned ***(no majority response)***.  **Survey 2**: 81% agreed with our suggestion to invite authors to report in the “methods” whether subgroup analyses were preplanned and provide guidance on how to report subgroup differences in the explanatory document ***(majority response)***. |
| *Rationale for change* |  | Test accuracy may vary across subgroups but many diagnostic accuracy studies lack the power to detect such variations. Multiple subgroup analyses can increase the risk of false-positive findings, and therefore it is more important to know whether they were pre-specified. |
| STARD 2003 | 24 | Report estimates of test reproducibility, if done. |
| STARD 2015 | 10a | Index test, in sufficient detail to allow replication |
|  | 10b | Reference standard, in sufficient detail to allow replication |
| *Survey results* |  | **Survey 1**: 80% agreed with our suggestion to remove the item and integrate it with the item about “technical specifications” (item 8 on the STARD 2003 list) **(*majority response*)**. |
| *Rationale for change* |  | The word “reproducibility” is ambiguous. Estimating a test’s reproducibility is not an element of most diagnostic accuracy studies. Many studies refer to other publications or to the manufacturer for information on test reproducibility, but this information should be part of the item about test specifications. |
| STARD 2003 | 25 | Discuss the clinical applicability of the study findings. |
| STARD 2015 | 27 | Implications for practice, including the intended use and clinical role of the index test |
| *Survey results* |  | **Survey 1**: 44% suggested to modify the item, while 27% preferred to keep the item as it is, and 27% suggested to remove it **(*no majority response*)**.  **Survey 2**: 34% agreed with our suggestion to modify the item and invite authors to adopt a structured discussion, while 47% preferred to keep the item as it is, and 13% suggested to remove it ***(no majority response)***. |
| *Rationale for change* |  | The original item was rather vague, general and not specific for diagnostic accuracy studies. Many reports of test accuracy studies offer generous and optimistic interpretations of the study findings, with strong recommendations for practice and therefore, more guidance is needed. Survey: no consensus could be reached on whether to keep this item as it is (47%) or adopt a structured discussion (34%). In line with CONSORT, it was decided to provide more structure by modifying the existing item, in which authors are now invited to report on implications for practice, and by adding a new item (item 26, see “Rationale for new items in STARD 2015” table below), in which authors are invited to report on study limitations. |

**2. Rationale for new items in STARD 2015**

|  | **Item #** | **Item description** |
| --- | --- | --- |
| STARD 2015 | 2 | Structured summary of study design, methods, results, and conclusions (for specific guidance, see STARD for Abstracts) |
| *Survey results* |  | This item was not addressed in the survey; but during the live meeting in September 2015, the STARD Steering Committee decided that guidance for the reporting for abstracts should be provided as well. |
| *Rationale* |  | Many journal and conference abstracts of diagnostic accuracy studies lack crucial study information. |
| STARD 2015 | 3 | Scientific and clinical background, including the intended use and clinical role of the index test |
| *Survey results* |  | **Survey 1**: 79% agreed with our suggestion to invite authors to report the purpose, clinical context, and clinical role of the test **(*majority response*)**. |
| *Rationale* |  | Many diagnostic accuracy studies report vague and very general study questions/aims. Prespecifying study hypotheses should be stimulated. This item is also part of CONSORT. |
| STARD 2015 | 18 | Intended sample size and how it was determined |
| *Survey results* |  | **Survey 1**: 78% agreed with our suggestion to add an item that addresses sample size considerations **(*majority response*)**. |
| *Rationale* |  | Sample size calculations are rarely reported in diagnostic accuracy studies. |
| STARD 2015 | 26 | Study limitations, including sources of potential bias, statistical uncertainty, and generalisability |
| *Survey results* |  | **Survey 2**: 34% agreed with our suggestion to modify the item and invite authors to adopt a structured discussion ***(no majority response)***. |
| *Rationale* |  | Many reports of test accuracy studies offer generous and optimistic interpretations of the study findings, with strong recommendations for practice and therefore, more guidance is needed. This item is also part of CONSORT. |
| STARD 2015 | 28 | Registration number and name of registry |
| *Survey results* |  | **Survey 1**: 74% agreed with our suggestion to recommend reporting the trial registration number **(*no majority response*)**. |
| *Rationale* |  | In a survey, we found that 15% of diagnostic accuracy studies are currently registered, but registration numbers are often not reported, making it difficult to identify the registered record.  There is a movement towards more openness and transparency in health research in general. This is not specific for test accuracy studies.  Important to future proof STARD: these items will become increasingly important in the following years. This item is also part of CONSORT. |
| STARD 2015 | 29 | Where the full study protocol can be accessed |
| *Survey results* |  | **Survey 1**: 84% agreed with our suggestion to recommend reporting about the availability of the study protocol **(*majority response*)**. |
| *Rationale* |  | There is a movement towards more openness and transparency in health research in general. This is not specific for test accuracy studies. Important to future proof STARD: these items will become increasingly important in the following years. This item is also part of CONSORT. |
| STARD 2015 | 30 | Sources of funding and other support; role of funders |
| *Survey results* |  | **Survey 1**: 89% agreed with our suggestion to recommend reporting sources of funding **(*majority response*)**. |
| *Rationale* |  | There is a movement towards more openness and transparency in health research in general. This is not specific for test accuracy studies. Important to future proof STARD: these items will become increasingly important in the following years. This item is also part of CONSORT. |
